# Practical Dynamic Contrast Enhanced MRI in Small Animal Models of Cancer: Data Acquisition, Data Analysis, and Interpretation

**DOI:** 10.3390/pharmaceutics4030442

**Published:** 2012-09-19

**Authors:** Stephanie L. Barnes, Jennifer G. Whisenant, Mary E. Loveless, Thomas E. Yankeelov

**Affiliations:** 1 Institute of Imaging Science, Vanderbilt University, Nashville, TN, 37232-2310, USA; Email: steph.barnes@vanderbilt.edu (S.L.B.); j.whisenant@vanderbilt.edu (J.G.W.); mary.loveless@vanderbilt.edu (M.E.L.); 2 Department of Radiology and Radiological Sciences, Vanderbilt University, Nashville, TN 37232, USA; 3 Program in Chemical and Physical Biology, Vanderbilt University, Nashville, TN 37232, USA; 4 Department of Biomedical Engineering, Vanderbilt University, Nashville, TN 37235-1826, USA; 5 Department of Physics and Astronomy, Vanderbilt University, Nashville, TN 37235, USA; 6 Department of Cancer Biology, Vanderbilt University, Nashville, TN 37232-6838, USA; 7 Vanderbilt-Ingram Cancer Center, Vanderbilt University, Nashville, TN 37232, USA

**Keywords:** DCE-MRI, mouse, cancer, diffusion, FLT, FDG, FMISO

## Abstract

Dynamic contrast enhanced magnetic resonance imaging (DCE-MRI) consists of the continuous acquisition of images before, during, and after the injection of a contrast agent. DCE-MRI allows for noninvasive evaluation of tumor parameters related to vascular perfusion and permeability and tissue volume fractions, and is frequently employed in both preclinical and clinical investigations. However, the experimental and analytical subtleties of the technique are not frequently discussed in the literature, nor are its relationships to other commonly used quantitative imaging techniques. This review aims to provide practical information on the development, implementation, and validation of a DCE-MRI study in the context of a preclinical study (though we do frequently refer to clinical studies that are related to these topics).

## 1. Background and Motivation

After tumors reach approximately 1 mm in diameter, diffusion can no longer provide the nutrients necessary to maintain growth, causing tumors to begin recruiting blood vessels; the well-known process of angiogenesis [1]. Angiogenesis is a hallmark of tumor development and progression, and pathological angiogenesis results in vessels that are characteristically distinct from those formed during physiological angiogenesis. Specifically, the vasculature in pathological angiogenesis is highly permeable, fragile, non-hierarchical, and torturous compared to normal vasculature. Given the propensity of angiogenesis in tumor progression, many anti-tumorigenic drugs have been developed to target this process. A common target for anti-angiogenic drugs is the cytokine vascular endothelial growth factor (VEGF), which is implicated in both physiological and pathologic angiogenesis, and is overexpressed in many tumor types [2,3]. For example, bevacizumab (Avastin^®^), a humanized monocolonal antibody, inhibits VEGF and has been used clinically for various types of cancer, including colorectal cancer, metastatic breast cancer, and recurring gliomas [4,5,6,7,8,9,10]. Sorafenib and sunitinib are tyrosine kinase inhibitors. They act by preventing signal transduction during angiogenesis by acting against the internal phosphorylation site of VEGF receptors to prevent ATP binding [3]; clinical trials have indicated their propensity to modestly prolong median survival in a variety of cancers [11,12,13,14,15]. In addition to being a target of anti-cancer therapies itself, tumor vasculature is also the means by which more conventional therapies are delivered to the tumor cells themselves and, therefore, is fundamental to understanding tumor response to treatment.

In order to test the efficacy of anti-vascular drugs in both the preclinical and clinical settings, it is important to non-invasively, serially, and quantitatively evaluate their effects on tumor vasculature. Dynamic contrast-enhanced magnetic resonance imaging (DCE-MRI) has been shown to be sensitive to characteristics of vasculature such as vessel perfusion and permeability, as well as vascular and extravascular volume fractions [16,17]; thus, it has the potential to serve as an imaging biomarker of the response of tumors to anti-vascular therapies [18,19,20,21,22,23,24,25,26]. These studies have demonstrated that DCE-MRI has the potential to become an accepted non-invasive indicator of tumor vascularity and therefore, ultimately, a biomarker of treatment response.

The goal of this contribution is to provide a survey of the current state of DCE-MRI in preclinical models of cancer and to provide a practical examination of the methodology of DCE-MRI. Furthermore, as multi-modality imaging becomes more common (see, e.g., [27,28,29,30,31]), we highlight several imaging metrics (particularly, positron emission tomography methods) that could prove to be complimentary to the data returned from a quantitative DCE-MRI study.

## 2. Basic Theory of DCE-MRI

### 2.1. Calibrating the Concentration of Contrast Agent to Measured MRI Parameters

Implementation of DCE-MRI entails the pharmacokinetic characterization of an injected contrast agent (CA) as it enters and exits the region under investigation (e.g., an individual voxel or a region of interest (ROI) within a tumor). Unlike CT or X-ray contrast agents, for which there is a linear relationship between the concentration of CA and the measured signal, MRI CAs indirectly alter the signal by shortening the native relaxation times of (most commonly) the hydrogen nuclei in water. In particular, DCE-MRI is concerned with the effect on the longitudinal relaxation time, *T*_1_. (Dynamic susceptibility contrast enhanced MRI, DSC-MRI, is concerned with *T*_2_ and *T*_2_^*^ effects and the interested reader is referred to [32].) The ability of a CA to enhance relaxation is quantified by its “relaxivity”, which describes how the *R*_1_ (= 1/*T*_1_) relaxation rate of the tissue is changed with respect to CA concentration; the relaxivity is a CA-specific parameter that also depends on the field strength employed in the study. The relaxivity may also vary for different tissue types as a function of tissue protein content [33]; however, current work in the field generally assumes a constant relaxivity value for a given CA, field strength, and temperature. The most common transformation from CA concentration to tissue *R*_1_ is given by Equation 1 [34]:

*R*_1_ = *r*_1_ · [*RA*] + *R*_10_ (1)

where *R*_10_ is the baseline relaxation of the tissue, [*CA*] is the concentration of the CA in the tissue, and *r*_1_ is the CA-specific relaxivity (in units of mM^−1^ s^−1^). (We note that Equation 1 is a “fast exchange limit” relation and assumes a linear relationship between CA concentration and *R*_1_; for more information on this somewhat controversial point, the interested reader is referred to, e.g., references [35,36,37,38]. We revisit this point in Section 6.) The effect of the CA results in an increase in the tissue signal intensity, to a degree determined by the accumulation of CA, on a *T*_1_-weighted imaging sequence. Dynamic acquisition of *T*_1_-weighted images before, during, and after CA administration allows for the generation of a signal intensity time course from a tissue of interest which can then be analyzed qualitatively or quantitatively to provide characterization of various features of that tissue’s physiology. 

When discussing ^1^H MRI, the effectiveness of a CA lies in its ability to alter the rate of relaxation of the hydrogen nuclei within the water molecules of the tissue of interest. This interaction is largely determined by the number of water molecules in the coordination shell, exchange rate of the coordinated and bulk water, the number of unpaired electrons in the CA, and the rotational correlation time of the CA. While details of this theory are presented elsewhere [39], here we merely note that the most commonly used MRI contrast agents are composed of gadolinium (Gd), which has seven unpaired electrons in its outer shell, within an appropriate chelate; gadolinium diethylenetriaminepentaacetic acid (Gd-DTPA) is one such molecule. Gd-DTPA is a small molecule (~0.6 kDa), stable *in vivo*, displays rapid clearance, and has shown minimal toxicity, and thus is used frequently in clinical (and preclinical) applications [40]. Though Gd-based CAs are generally considered quite safe, associations have recently been made between such agents and Nephrogenic Systemic Fibrosis (NSF) [41]; ongoing research is investigating the relationship between Gd-based CAs and this disease [42,43,44,45,46]. We note that the relaxivity of Gd-DTPA is on the order of 4 mM^−^^1^ s^−^^1^ at 1.5 T [47] and that the design of more effective CAs is a field of active research [48]. In this review, we focus on the commonly used small molecule Gd-chelates; the interested reader is referred to references [49,50,51] for discussions of other CAs, including macromolecular CAs.

### 2.2. Classes of DCE-MRI Methods

DCE-MRI is actually a class of techniques in which the individual approaches are characterized by whether they provide qualitative, semi-quantitative, or quantitative data. Qualitative and semi-quantitative analysis of DCE-MRI data examine characteristics such as maximum uptake of the contrast agent, wash-in/out rates, and the area under the signal intensity curve [52], while quantitative analysis of the data requires modeling of the signal intensity curve to extract physiologically-related parameters. Methods from all three categories have been employed for tissue curve analysis and have been shown to correlate with treatment response [20,21,53,54,55,56,57,58,59,60]. There are distinct advantages and disadvantages associated with all three types of analysis, based mainly on the data acquisition required and the specificity of the evaluated information.

#### 2.2.1. Qualitative Methods

**Figure 1:**
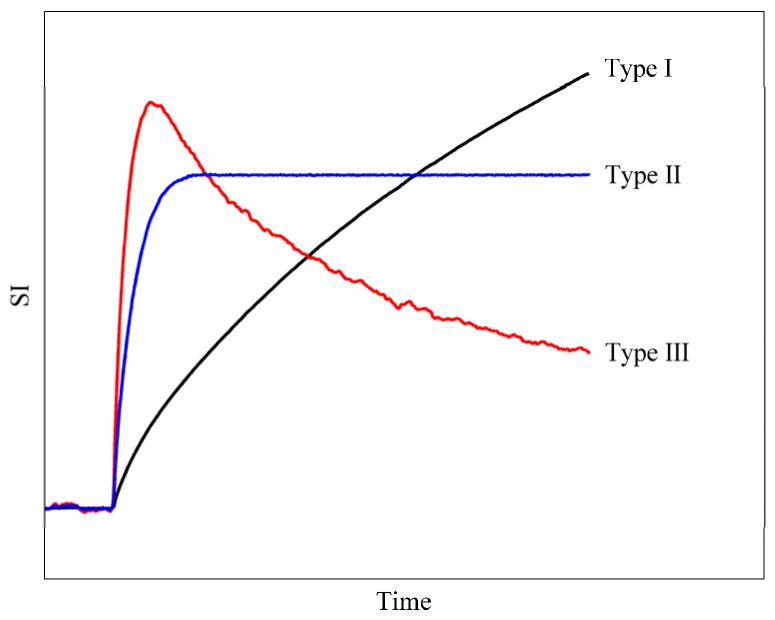
Three illustrative curve shapes for qualitative Dynamic contrast enhanced magnetic resonance imaging (DCE-MRI) analysis. The type III curve consists of a sharp uptake, followed by a distinct washout and is frequently a sign of malignancy (see, e.g., breast cancer). The type II curve may have a similar uptake, but the rapid washout is absent. The type I curve demonstrates gradual, continual uptake over the course of the experiment.

Qualitative analysis relies on an evaluation of the signal intensity curve behavior in the voxel or ROI. The shape of the curve is typically placed into one of three general categories: type I, type II, or type III [61,62,63,64]. A type III shape is defined by the decrease in signal intensity after the peak signal intensity achieved during the initial phase of the curve. Type II curves display a signal intensity that remains relatively constant in time after the initial peak. The final category, type I, defines a curve in which the signal intensity continues to increase during the acquisition time. Representative curves for each of the three categories are shown in Figure 1. The advantage to qualitative analysis is that the only necessary component is the dynamic signal intensity curve; in particular, qualitative analysis does not necessitate acquisition of the pre-contrast *T*_1_ map (Section 2.3) or knowledge of the arterial input function (AIF - Section 2.5). However, a major disadvantage to a qualitative analysis is that it does not provide quantitative parameters that are directly related to the underlying physiological tissue characteristics. It also makes comparisons between results achieved at different sites difficult because signal intensity has no physical units and can be influenced by technical image acquisition parameters. However, this does not eliminate the benefit of qualitative analysis, as evaluation of curve shape has been able to discriminate, e.g., between benign and malignant breast tumors [64,65]. (It should be noted that qualitative assessment of tumor morphology is often used as an adjunct to DCE analysis, and, in fact, can be improved by assessing the morphology of the enhancing region in a DCE acquisition; the interested reader is referred to, e.g., [66,67,68,69].).

#### 2.2.2. Semi-Quantitative Methods

**Figure 2:**
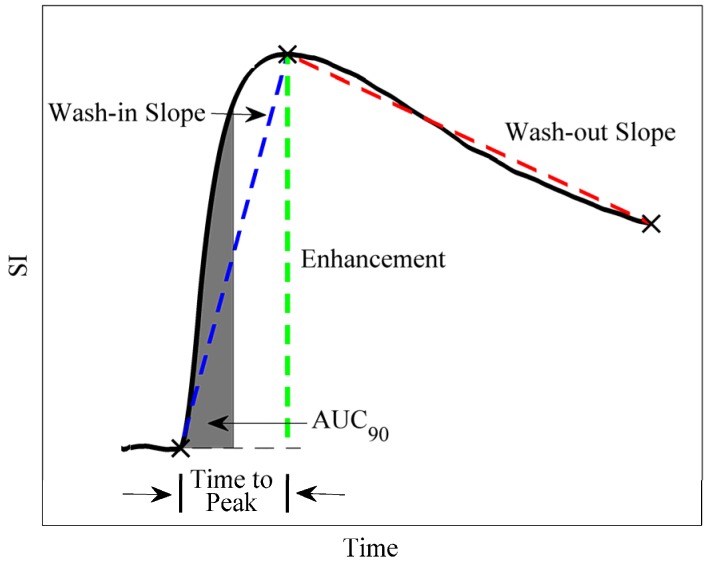
The figure depicts several parameters that are commonly explored in semi-quantitative DCE-MRI analysis. The black line shows a representative signal intensity curve. The gray shaded area indicates the initial area under the curve (AUC) for the first 90 s after injection of the contrast agent. The blue dashed line represents the wash-in slope and the red dashed line represents the wash-out slope. The green dashed line shows the enhancement. Finally, the bars between the arrows indicate the time to peak.

Semi-quantitative analysis consists of a group of parameters that require calculation based on curve properties. Typical semi-quantitative values are area under the curve (AUC), enhancement, time to peak, and wash-in/wash-out slope [63,70] as illustrated in Figure 2. The initial AUC is calculated as the area under the voxel or ROI signal intensity (or concentration, if the pre-contrast *T*_1_ map is obtained) curve from the time of injection to some designated time post-injection, usually 60 or 90 s. The enhancement is quantified as the change in signal intensity from baseline, divided by the baseline signal intensity value. Time to peak is designated as the time from injection of the CA to the maximum of the signal intensity curve. The wash-in and wash-out slopes are defined as the slope of the dynamic curve from the point of injection to the peak of the curve, and the slope from the peak until the end of acquisition, respectively. The benefits and disadvantages related to semi-quantitative analysis are similar to those in the qualitative analysis. Namely, benefits of a semi-quantitative DCE-MRI approach include the less complicated and time consuming acquisition requirements (e.g., semi-quantitative methods do not require AIF measurement) and the ease with which the post-processing can be accomplished. Similar to qualitative approaches, the disadvantages of semi-quantitative approaches include that it is difficult to directly relate these measures to underlying physiology, and to compare results achieved at different sites. However, broad correlations can be established between semi-quantitative parameters and underlying physiology. For example, increased vascular density and/or vascular permeability will likely increase the wash-in slope, AUC, and peak enhancement, while decreasing the time to peak. Additionally, the cell density of the region can affect the wash-out slope by decreasing the available space for CA to pool. Semi-quantitative methods have been used to assess the effect of anti-vascular drugs in clinical [55,56] and preclinical settings [21,57,58,59]. For example, Robinson *et al.* utilized the AUC for the first 150 s after injection to evaluate the effect of an anti-vascular agent on tumor perfusion in a rat model of thyroid cancer [21]. The results showed a marked shift in the histogram of voxel-based AUC values after treatment, indicating a decrease in tumor perfusion. Marzola *et al*. used the AUC to evaluate the effect of a tyrosine kinase inhibitor in a murine model of colon cancer [58]. The results showed a significant decrease (*p* < 0.05) in the AUC (92 s) between the pre-treatment and 24 h post-treatment acquisitions. Tang *et al.* used the AUC (90 s) to determine the effect of tumor necrosis factor α (TNF-α) on tumor microvasculature of colon adenocarcinomas implanted on the hind limb of mice [59]. The authors found a significant decrease in the AUC at 6 h and 96 h post treatment.

#### 2.2.3. Quantitative Methods

**Figure 3:**
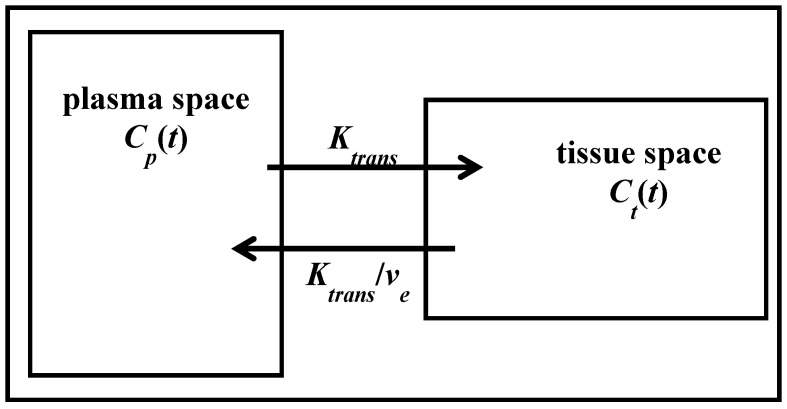
Two compartment model showing one compartment representing the plasma space while the other compartment is the tissue space. The contrast agent leaves the plasma space at a rate represented by *K^trans^* (the volume transfer constant) and returns by *K^trans^*/*v_e_* (the efflux constant).

In standard quantitative DCE, the dynamically acquired tissue curves can be fit to appropriate mathematical models in order to obtain quantitative parameters that directly reflect physiological parameters such as tumor vessel perfusion and permeability and tissue volume fractions. The most commonly used model is frequently referred to as the Kety-Tofts model in which the concentration of CA is considered in just two compartments, the blood/plasma space (denoted by *C_p_*) and the tissue space (denoted by *C_t_*), as shown in Figure 3 [16,71]. DCE-MRI notation was standardized by Tofts *et al*. in 1999, where *K^trans^* represents the volume transfer constant from the plasma space to the tissue space and *v_e_* is the extravascular-extracellular volume fraction [16]. It is important to note that *K^trans^* has different physiologic interpretations depending on factors such as permeability and blood flow for the tissue of interest. This process can be described in four ways: (1) flow limited (areas with high permeability); (2) permeability-vessel surface area (*PS*) limited (areas with low permeability); (3) mixed flow and PS (where neither flow nor permeability is the main limiter, but rather a combination of the two); and (4) clearance (removal of the CA from the tissue compartment) [16].

As Tofts *et al.* describes, if a homogeneous distribution of CA is assumed in both compartments, then the concentration change within the tissue compartment can be described by a linear first order ordinary differential equation:

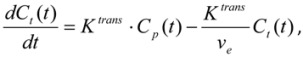
(2)
the solution to which is given by:

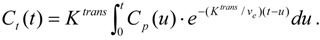
(3)
This model neglects any fraction of the tissue that may contain vascular space; however, investigators have shown that this fraction may not be negligible in several types of cancer [72,73,74]. Therefore, Equation 3 has been amended to include a third parameter to reflect the fraction of vascular space (*v_p_*) as a part of the tissue space and is described by [74]:

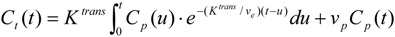
(4)

In these forms, if *C_p_*(*t*) and *C_t_*(*t*) are measured, then the data and Equation 3 or 4 can be put into a curve fitting routine to extract estimates of *K^trans^* and *v_e_* (and *v_p_*) for the tissue data on a voxel-by-voxel or ROI basis. A point to consider when performing quantitative DCE-MRI analysis is that different optimization schemes can result in different parameter estimates, and hence different software packages may produce different results; thus, the same software should be utilized for all analyses within a study.

Of the three classes of DCE-MRI analysis, this approach has the clearest correlation to underlying physiology. Quantitative parameters have been used to successfully monitor cancer treatment over time and have been shown to reflect histological changes in tumor vasculature [20,60,75,76,77,78]. Maxwell *et al.* utilized quantitative DCE-MRI to evaluate the effect of a vascular-inhibiting drug in carcinosarcomas in rats and found that *K^trans^*, which decreased post-treatment, corresponded closely (*r* = 0.98, *p* < 0.001) to the change in blood flow as measured by radiolabelled iodoantipyrine uptake [20]. Checkley *et al.* investigated the effect of a VEGF signal inhibitor on prostate adenocarcinoma xenografts using DCE-MRI [60]. The work indicated decreases in mean *K^trans^* and *v_e_* for all doses, with significant decreases in *K^trans^* for doses ≥25 mg/kg and in *v_e_* for doses ≥50 mg/kg. However, though quantitative parameters have physiological meaning, their interpretation is not always obvious *a priori*; in particular, *K^trans^*, as noted above. Additionally, quantitative analysis requires the most complex and time consuming acquisition, as it is necessary to obtain both a measure of the pre-contrast *T*_1_ values and a measure of the AIF, which raises particular challenges in small animal imaging. Another important consideration is that quantitative parameters can be sensitive to errors related to, for example, *T*_1_ mapping and AIF characterization. Further, increasing the complexity of the analysis can potentially increase the error in the estimated parameters. For example, a more complex model (e.g., the extended model as compared to the standard model) with more free parameters will inherently have more error in the parameterization.

### 2.3. Pre-Contrast *T_1_*

Implementation of quantitative DCE-MRI (and semi-quantitative analysis, when the curve is converted to concentration) requires the native *T*_1_ of the tissue (*T*_10_). As there is typically not enough time to acquire a *T*_1_ map at each serial acquisition during and after the injection of CA, the pre-contrast *T*_1_ map is critical for calibrating the signal change due to the entrance and exit of the contrast agent to the time dependent concentration of the CA in the tissue, *C_t_*(*t*). Rapid and accurate determination of *T*_1_ values is required, but can be quite difficult to obtain in practice. Fortunately, there are several methods regularly employed in small animal studies for determining the *T*_1_ of tissue [79]. *T*_1_ can be measured using a standard spin echo sequence to collect several sets of images at a constant *TE* and a varied *TR*. These image sets are then fit to the spin echo signal intensity equation to estimate a value for *T*_1_ on a voxel-by-voxel or ROI basis. A common variation to this method is the inversion recovery spin echo sequence which acquires multiple images at a set of varied inversion times (*TI*, delay time after inversion pulse), followed by fitting the data to the appropriate signal intensity equation to estimate *T*_1_. Generally, these methods are long (approximately one hour) and not convenient for many *in vivo* studies. One method to decrease acquisition time is to incorporate fast imaging readouts (e.g., echo planar); however, susceptibility distortions limit *in vivo* applications at high field strengths [80]. Alternatively, studies have employed a snapshot FLASH (fast low angle shot) technique, which uses many low flip angle acquisitions to collect a signal recovery curve following an inversion pulse [81,82]. However, depending on the number of *TI*’s, acquisition time can be greater than 25 min. A very popular method, especially in clinical applications, is a spoiled gradient recalled echo (SPGR) sequence with a fixed *TR* and *TE* and a varied flip angle [59,83,84,85]. This method can be used when short acquisition times (<10 min) and/or large volumetric coverage is required [86]. However, the accuracy of this method largely depends on radiofrequency uniformity, which is well-known to decrease with increasing field strength. Thus, it is recommended to acquire a separate imaging sequence to map the flip angle and correct for any imperfections [87,88]. 

### 2.4. Requirement of Fast *T_1_* Imaging

Acquisition of the *T*_1_-weighted images before, during, and after CA injection must occur rapidly in order to provide the necessary temporal resolution to observe the effect of the CA. However, as is the case with all MR imaging, temporal resolution is directly linked to spatial resolution, field of view (FOV) and signal-to-noise ratio (SNR). In oncologic applications, it is generally important to cover as much of the tumor volume as possible; in addition, the acquisition must be rapid enough to characterize the varying CA kinetics in the heterogeneous tumor region. Typical temporal resolutions can range from 1–30 s, depending on the application. If mapping tumor heterogeneity by modeling tumor CA kinetics on a voxel-by-voxel basis is desired, then spatial resolution must be high enough to differentiate the details of the lesion. However, increasing the spatial resolution necessarily limits the temporal resolution and signal-to-noise of the acquired data. Thus, the relative importance of temporal resolution, spatial resolution, and SNR is dependent on the goals of the study. For this reason, most studies employ a heavily *T*_1_-weighted SPGR sequence with a minimum *TR* (to maximize temporal resolution) and minimum *TE* (to minimize *T*_2_^*^ effects). Unfortunately, fast *T*_1_ imaging frequently necessitates a low spatial resolution which can create problems for characterizing the AIF from small diameter vessels (e.g., linguofacial artery). Table 1 summarizes illustrative studies with typical sequence parameters where high temporal resolution was chosen over spatial resolution or vice-versa.

**Table 1:** Sequence parameters for studies designed to obtain either high spatial resolution or high temporal resolution.

Author, year [reference]	Spatial resolution	Temporal resolution	Description
Loveless, 2012 [89]	in-plane = 0.27 mm^2^; matrix = 128^2^; slice thickness = 1 mm	temporal resolution = 25.6 s; *TR*/*TE*/α = 100 ms/2.82 ms/25°	Used a population average AIF since assessing heterogeneity from whole tumor volume was a priority. Study sacrificed temporal resolution for high spatial resolution.
Benjaminsen, 2004 [90]	in-plane = 0.5 mm × 0.2 mm; matrix = 256 × 128; slice thickness = 2 mm	temporal resolution = 27 s; *TR*/*TE*/α = 200 ms/3.6 ms/80°	Used blood sampling to determine AIF, and sacrificed temporal resolution for whole tumor volume coverage. Also used a population average AIF from the left ventricle of additional animals with different scan parameters to achieve a faster temporal resolution.
Kim, H 2011 [91]	in-plane = 0.23 mm^2^; matrix = 128^2^; slice thickness = 1 mm	temporal resolution = 58.8 s; *TR*/*TE*/α = 115 ms/3 ms/30°	Used a reference region analysis since spatial resolution and whole tumor volume coverage was a priority. Study sacrificed temporal resolution for high spatial resolution.
Li, 2010 [92]	in-plane = 0.35 mm^2^; matrix = 128 × 64; slice thickness = 1 mm	temporal resolution = 1.6 s; *TR*/*TE*/α = 25 ms/1.4 ms/20°	Used a fast gradient echo sequence to achieve high temporal resolution in order to collect individual AIFs from image data. Study sacrificed whole tumor volume coverage by only collecting three slices.
Skinner, 2012 [93]	in-plane = 0.25 mm^2^; matrix = 128^2^; slice thickness = 2 mm	temporal resolution = 1.9 s; *TR*/*TE*/α = 10 ms/2.1 ms/15°	Used individual AIFs for kinetic modeling. Study sacrificed whole tumor coverage (only collected central tumor slice) to achieve high temporal resolution.
Kim, J 2012 [94]	in-plane = 0.23 mm^2^; matrix = 128^2^; slice thickness = 2.5 mm	temporal resolution = 6.4 s; *TR*/*TE*/α = 67 ms/3 ms/70°	Used fast imaging sequence to achieve temporal resolution, however study sacrificed through-plane spatial resolution (2.5 mm) for whole tumor volume coverage. AIF was collected from image data for kinetic modeling, although 6.4 s might be too long to adequately sample the peak of the CA concentration curve.

### 2.5. AIF

**Figure 4:**
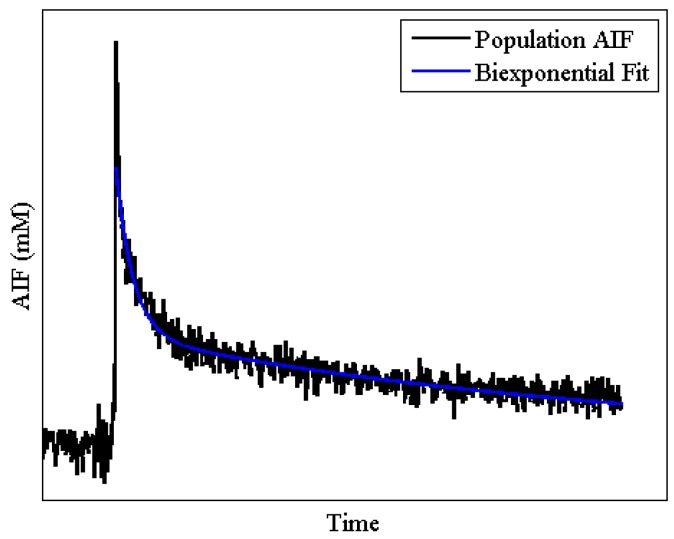
Typical AIF in a mouse system. The black line represents a population AIF obtained in mice. Notice the rapidness with which the peak occurs. The blue line shows a standard biexponential fit to the washout portion of the curve.

The time rate of change of the concentration of the CA in the blood pool (*C_p_*(*t*), the AIF) is required for most quantitative DCE-MRI modeling approaches. The AIF is characterized by a sharp wash-in followed by a short-lived peak concentration and subsequent longer wash-out period. Because the AIF kinetics are much more rapid than tissue kinetics, it is often difficult to optimize the temporal resolution required to not only well-characterize the AIF, but also obtain a desirable SNR and spatial resolution in the measurement of tumor tissue kinetics. Additionally, location of a blood pool large enough to be useful in AIF characterization does not always coincide with the FOV required to gather the kinetic data within the tumor. Using a population derived AIF or reference tissue have been proposed as alternatives to acquiring an individual AIF for each subject, a point we will return to in Section 3.1 [81,95]. An example of a typical AIF for a mouse system is illustrated in Figure 4.

## 3. Specific Considerations for Small Animal Imaging

### 3.1. AIF Measurement

As mentioned previously, the components required for a DCE-MRI study will vary depending on the overall objective of the experiment. In the case of a study requiring a quantitative analysis of DCE-MRI data, some estimate of the AIF is required. Acquisition of the AIF requires special consideration in preclinical applications utilizing small animal (commonly rodent) subjects. In small animals particularly, the concentration of CA in the blood plasma changes very rapidly in comparison to tissue kinetics; this is of concern because it is imperative to capture the peak of the AIF signal or important information regarding the kinetics of the tissue will be lost. There are three main ways for estimating the AIF: blood sampling, from the images themselves, and forming a population average. Unfortunately, each of these methods has their own set of limitations.

The gold standard for AIF characterization is blood sampling [96]; however, sampling is very invasive and in small animal imaging, where the subjects generally have a small blood volume (e.g., a mouse has a total blood volume of approximately 2 mL), the number of samples that can be collected is quite limited. Furthermore, the sampling is typically taken from a vessel far away from the tissue of interest and may not be an accurate indicator of what AIF the tumor actually experiences.

Alternatively, the AIF can be quantified non-invasively though imaging, wherein the signal from a blood source can be converted to CA concentration to give a direct evaluation of the AIF [97,98]. This method has the potential advantage of accurately measuring the AIF for each individual animal, and it is completely non-invasive. Loveless *et al.* recently developed a protocol to estimate the AIF from the left ventricle in mice [81]. A cardiac-gated and respiratory-gated gradient echo sequence was used to locate a 2 mm slice along the short-axis view of the left ventricle of the heart. The DCE-MRI protocol used a *T*_1_-weighted gradient echo sequence at a temporal resolution of 1.5 s with the following scan parameters: *TR*/*TE*/α = 6 ms/2.41 ms/10°; NEX = 4; FOV = 25 mm^2^; matrix = 64^2^. A 120 µL bolus of 0.05 mmol/kg Gd-DTPA was delivered via a surgically emplaced jugular catheter using an automated syringe pump (Harvard Apparatus, Holliston, MA) at a rate of 2.4 mL/min after 100 baseline images were acquired. Each voxel within the left ventricle was examined for specific characteristics to eliminate the potential effects of flow and motion artifacts, including SNR ≥ 30 at all dynamic time points and peak concentration ≥ 0.15 mM. All returned voxels were also visually inspected for appropriate washout activity to prevent potential myocardial wall contamination. It should be noted that using this protocol to collect the AIF from imaging data requires that the left ventricle (or other feeding vessel) be located in the FOV, which is often simply not possible given the location of the tumor. Additionally, quantification of the AIF requires a high temporal resolution that limits the SNR and/or the spatial resolution of the dynamic acquisition—all of which undermines the ability of DCE-MRI to accurately and quantitatively assess tumor heterogeneity.

Two common methods are utilized in small animal imaging to circumvent the issues surrounding collection of a subject-specific AIF. The first is using a population derived AIF [81,99,100,101,102,103]. In this approach, the average AIF is quantified for a group of subjects, and then is used in performing quantitative analysis of future, similar subjects. This eliminates the need to calculate a subject-specific AIF; since the CA tissue kinetics are much slower than in the blood, eliminating the collection of the subject AIF allows for a decrease in the temporal resolution and hence an increase in the SNR and/or spatial resolution of the dynamic data. Furthermore, the population based AIF, having been formed by averaging several individual AIFs, generally has quite high SNR. Of course, the main disadvantage to this approach is the potential for substantial variation between subjects. As the AIF is used as the driving factor for the models used in the quantitative analysis (see Equations 3 and 4), subject variability will result in parameterization error [81,103,104]. Loveless *et al.* not only standardized a protocol for collecting AIFs within the left ventricle of mice, they also compared the similarity of the parameters derived from quantitative DCE-MRI analysis using an individual versus a population AIF in a murine model of breast cancer [81]. In the case of the extended model analysis, and for Gd-DTPA, the authors found a concordance correlation coefficient (CCC) of 0.96 for *K^trans^*, 0.88 for *v_p_*, and 0.80 for *v_e_* for the ROI parameter values. On a voxel-basis, the CCC values for the individual mice ranged from 0.89 to 1 for *K^trans^*, 0.58 to 0.98 for *v_p_*, and 0.29 to 0.99 for *v_e_* [81]. However, the results can be confounding as a study by Pickup *et al.* found significant inter-subject variability in the measured AIF of mice and concluded that a subject specific AIF was necessary as opposed to a population AIF for accurate assessment [104]; though it should be noted, the latter was a limited study in a small group of animals. In order to obtain the most consistent results when utilizing a population AIF, it is imperative that the experimental protocol be rigorously defined and followed in order to ensure consistency between the population AIF and the individual subject acquisition, a point we return to in Section 3.2 and Section 3.4.

The second method that has been investigated as an alternative to acquiring an individual AIF is the “reference region” (RR) approach [95,105,106,107,108,109,110]. Analysis by the RR approach utilizes a well-characterized tissue such as muscle to calibrate the signal in the ROI. Using a RR approach eliminates the need to characterize the AIF; thus it is not necessary to have a blood pool in the FOV or to directly measure the AIF. In the integral form, the RR approach utilizes two copies of Equation 2: one for the tissue of interest, as given by Equation 2, and one for the reference region tissue:

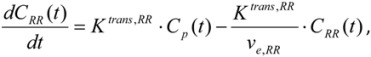
(5)
where *K^trans,RR^* and *v_e,RR_* are the appropriate quantitative parameters for the reference region tissue, *C_RR_* is the measured concentration of CA in the reference region tissue, and *C_p_* is the plasma concentration of the blood. The set of equations allows for elimination of *C_p_*, and the solution of the resulting differential equation is given by [95]:

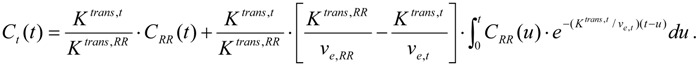
(6)

The RR approach was originally introduced by Kovar *et al.* [105]. The authors used the differential form of the equation to evaluate mammary adenocarcinomas and prostate tumors in rats. An estimation of the plasma concentration of CA was obtained from the muscle reference region using assumed values of the product of the perfusion rate and extraction fraction and extracellular volume. The plasma concentration was then used to optimize the values of the extracellular fraction and the product of the perfusion rate and extraction fraction in the tumor. The authors found that the errors in the tumor data fits produced by this approach were not significantly different than the noise level. Work by Yankeelov *et al.* evaluated the use of the RR model to characterize the tumor ROI [108]. The authors compared the *K^trans^* and *v_e_* values obtained from the RR analysis in nine rats to the *K^trans^* and *v_e_* values obtained using the standard Kety-Tofts model and the individual measured AIF, which was obtained using a combination of blood sampling and imaging of the aorta. The results indicated no significant difference between the two sets of parameters. The RR approach has its advantages in relative ease of image acquisition and in that it eliminates the need to characterize the AIF; however, if the reference region characteristics differ from those assumed from literature, or if the reference region is poorly characterized, the accuracy of the method decreases.

### 3.2. Reproducibility/Repeatability

In generating any protocol for use in preclinical and clinical studies, it is important to develop a rigorous experimental design in order to ensure reproducibility of the technique. A measure of institutional repeatability and reproducibility is beneficial in that it allows for comparisons across scans, and enables an analysis of a significant change in parameter, such as would be necessary to analyze, for example, treatment response data. Repeatability is defined as the variability in an individual person’s measurement of the same object, while reproducibility defines the inter-user variability. The available literature regarding the repeatability or reproducibility of DCE-MRI techniques is fairly limited [70,102,109,110,111,112]. The repeatability value is of particular interest as this defines the expected limit of the difference between two scans on the same subject in 95% of the cases. In other words, this value defines the difference between two scans that can be attributed to protocol and noise as opposed to physiological changes. Another parameter of interest is the 95% confidence interval (CI) of the mean, which is a measure of the reproducibility of the group mean parameter value. Galbraith *et al.* conducted a clinical study regarding the reproducibility of quantitative and semi-quantitative parameters in muscle and tumors [70]. *K^trans^* required a log_10_ transform in all cases due to the dependence of the difference between scans on the average scan magnitude. The repeatability values and the 95% CI for the quantitative analysis are presented in Table 2. The authors also investigated semi-quantitative parameters including AUC, gradient, and enhancement, and all parameters showed a decrease in the repeatability coefficient in the muscle as compared to the tumor.

In small animal imaging, the available literature regarding repeatability and reproducibility is very limited. Yankeelov *et al.* used the RR approach in a murine model of breast cancer to assess repeatability of the protocol [109]. The authors assumed *v_e_* of muscle, and fit *K^trans^* of muscle (the reference region) and *K^trans^* and *v_e_* of the tumor. Barnes *et al.* performed an investigation of quantitative DCE-MRI parameters in a murine model of breast cancer using a population AIF [113]. The work was performed on the median values for both a 64^2^ acquisition and a 128^2^ acquisition and using a center slice analysis and a whole tumor analysis. Again, the repeatability indices and 95% CI on the mean for the aforementioned studies are presented in Table 2.

**Table 2:** Dynamic contrast enhanced magnetic resonance imaging (DCE-MRI) reproducibility data for human and small animal studies.

Author, year [reference]	Subject	Tissue	Parameter	95% CI	Repeatability Index
Galbraith, 2002 [70]	Human	Tumor Muscle	*K^trans^*	(−16%)–(+19%)	0.32 mL(blood)/mL(tissue)/min
			*k_ep_*	±16%	0.91 mL(blood)/mL(tissue)/min
			*v_e_*	±6%	7.62 mL/mL
			*K^trans^*	(−30%)–(+44%)	0.61 mL(blood)/mL(tissue)/min
			*k_ep_*	±61%	1.28 mL(blood)/mL(tissue)/min
			*v_e_*	±13%	5.71 mL/mL
Yankeelov, 2006 [109]	Mouse	Tumor Muscle	*K^trans^*	*	0.222 mL(blood)/mL(tissue)/min
			*v_e_*		0.204 mL(blood)/mL(tissue)/min
			*K^trans^*		0.197 mL/mL
Barnes, 2012 [113]	Mouse	Tumor	*K^trans^*	±14%	0.073 mL(blood)/mL(tissue)/min
			*v_e_*	±8%	0.113 mL/mL
			*K^trans^*	±21%	0.075 mL(blood)/mL(tissue)/min
			*v_e_*	±5%	0.069 mL/mL
			*v_p_*	±15%	0.014

*kep* = *Ktrans*/*ve*; * data not included in original paper.

### 3.3. Animal Care and Monitoring

In utilizing small animals for a DCE-MRI study, there are details regarding care and monitoring which must be considered. For example, small animals will need to be anesthetized during imaging sessions in order to eliminate movement and minimize stress on the animal. Thus is it necessary to either give an appropriate dose of an injectable anesthetic prior to imaging, or to have an anesthesia delivery system that is MR-compatible and will provide a controllable amount of anesthesia to the animal throughout the study. The necessity for anesthesia also imposes limitations on the duration of the study. If an injectable anesthetic is given, then the duration of the scan will be dictated by the dose of the anesthetic. Additionally, if the imaging session is a survival experiment, one will need to consider the fact that the longer an animal is under anesthesia the harder it will be for the animal to wake. Typically, scans on the order of three to four hours are the maximum manageable limit for small animals. Selection of anesthesia agent may also be an important consideration. Though the literature is limited, a study regarding PET imaging showed that an injectable anesthesia (ketamine) had different effects on the imaging than did an inhalable anesthesia (isoflurane) [114]. Specifically, the work showed that ketamine caused a significant increase in the serum glucose levels whereas isoflurane did not. The effect of anesthesia is a detail of small animal DCE-MRI that requires further investigation.

While the animal is under anesthesia, it is useful to be able to monitor the animal’s heart and/or respiratory rate. The benefit here is two-fold: first, monitoring one of these rates serves as a secondary indication of the depth of anesthesia of the animal. If the rates are too high, the anesthesia may be too light; conversely, if the rates are too low or erratic, the anesthesia may be too high. Secondly, in regards to the previous discussion on consistency of the protocol (Section 3.2), it is logical to maintain a similar heart rate between animals, especially when utilizing a population AIF, which depends on rigorous repetition of the experimental setup. A drastically different heart rate will affect the blood flow and hence may have implications on the delivery of the CA during DCE-MRI, which could impact the use of the population AIF. Devices and hardware have been developed which are MR-compatible and can monitor respiratory and heart rates of small animals. The respiratory rate can be measured via a respiratory pillow braced against the animal’s abdomen such that expansion of the abdomen during inhalation compresses the pillow. The heart rate is generally measured using two probes placed subcutaneously on the two front paws of the animal; these probes must be well-secured to ensure that the electrocardiogram (ECG) measurement is not compromised by respiratory movement. Another important consideration in small animal imaging is the monitoring of body temperature. Small animals, specifically rodents, do not regulate their body temperature when under anesthesia. Thus it is necessary to monitor their body temperature and provide a heating source in order to regulate the temperature. Temperature monitoring is usually achieved by a probe that is either inserted in the rectum or placed in close vicinity to the animal. Temperature regulation is often accomplished by means of heating pads when prepping the animal, and through forced air down the MR bore while scanning. 

Other considerations for small animals include the administration of the CA and blood sampling, if desired. Both of these generally require the insertion of a catheter, though it is possible to inject the CA intraperitoneally so as to avoid catheterization; however, it is important to note that this approach obfuscates the ability to perform a quantitative DCE-MRI study. In small animals, catheters are typically inserted in either the jugular or tail vein. The jugular vein catheter is useful for longitudinal studies as it can be placed out of reach of the animals such that the animal cannot chew on it, and thus it can remain in place over the course of the experiment, provided the study is (maximally) no longer than 14 days. Use of the tail vein catheter can be quite difficult because, in small animals, the tail vein can be very small and very difficult to localize, thus making catheter insertion challenging. Additionally, a tail vein catheter can be inconvenient for longitudinal studies as it must be removed after imaging and replaced for subsequent imaging scans since the animal can reach the catheter and will affect it. Furthermore, it is not uncommon for this procedure to “collapse” the vein which makes future insertions challenging. Catheterization of the animal allows for CA injection directly into the circulation, which is central to DCE-MRI. However, the catheter requires care in order to ensure that it remains patent. This includes flushing of the catheter, generally daily, with saline or heparinized saline to prevent clotting. This is especially important in longitudinal or repeat studies where the catheters need to be maintained for a prolonged period of time (though generally speaking, in small animals, catheters can only be maintained for approximately 10–14 days). Since DCE-MRI depends on the delivery of the CA to the circulation, and subsequently to the tissue of interest, patency of the catheter is imperative to maintain delivery efficacy. 

### 3.4. Planning a Small Animal DCE-MRI Study

In designing a preclinical DCE-MRI study, there are a several key considerations that should be addressed prior to acquiring data. The main point to consider is the goal of the study, as this will often determine other factors. As with any MR imaging protocol, trade-offs exist between temporal resolution, spatial resolution, and SNR. So, for example, if the purpose of the study is to address rapid temporal changes, then high temporal resolution data (~1 s – 2 s) should be acquired. If, on the other hand, the experiment must assess changes in tissue physiology at the voxel level, then high spatial resolution should be acquired and this may necessitate a reduction in temporal resolution. Thus, the size of the imaging voxel that will be required (*i.e.*, what spatial scale is to be probed?) needs to be selected; this, in turn, will determine the upper limit on the achievable temporal resolution. Additionally, changing the spatial and/or temporal resolution will affect the SNR of the acquired data, and vice-versa. Therefore, given the SNR demands of the DCE-MRI analysis to be performed (which can be acquired through, for example, simulations), the relative importance of temporal resolution, spatial resolution, and SNR for a particular application must be considered in context of the goals of the study.

Another factor that is fundamentally related to the available SNR is, of course, the field strength of the magnet. Thus, prior to imaging, decisions must be made regarding optimal field strength. Obviously this will be at least partially dictated by the magnets available at the institution at which the study is being performed. However, given an option between field strengths, higher field strengths correspond to increased SNR. An inherent problem with higher field strengths, though, is the increased presence of ***B***_0_ field inhomogeneity, which can be of particular concern in gradient echo sequences, and artifacts. Thus, in making the selection of field strength, one should consider the desired scan parameters and compare this to the acceptable limit of field inhomogeneity and artifact presence. Going to higher fields also leads to longer *T*_1_ values and shorter *T*_2_ values; the former makes several quantitative *T*_1_ mapping procedures more time consuming, and the latter partially negates the increase in SNR available at higher fields. (There is a mature literature on this topic and for an introduction to the field, the interested reader is referred to [115].) The user will also need to consider the RF coil which will be utilized for the scan. Generally, DCE-MRI acquisitions are performed using volume coils, as opposed to surface coils, due to the limited acquisition volume associated with surface coils and the increased homogeneity present in volume coils. In selecting the coil, the size of the animal will be the dominating factor. The coil should be large enough to fit the animal at the region of interest; further, contact of the animal with the coil should be avoided. However, though the animal needs to fit in the coil, signal is maximized when the animal takes up as much space as possible within the coil. Typical coils sizes for mice included 25 mm and 38 mm coils, while a common size for rats is 63 mm for body imaging (though, the 38 mm coil can be used for some rat brain studies). Thus, coil selection will be at least partially determined by the smallest coil into which the animal comfortably fits.

In considering a quantitative DCE-MRI acquisition, the parameters depend not only on the image acquisition protocol, but also the delivery of the CA, and the method of estimating the AIF. Varying the way in which the CA is delivered or the timing of the CA delivery (*i.e.*, bolus injection and injection time) will necessarily affect the observed tissue kinetics. For this reason, it is important to have consistency in the delivery of the CA. This can be achieved by use of an automatic injector or by very strict protocols regarding manual injection, though the latter will almost certainly reduce the reproducibility of any DCE-MRI technique. Additionally, in reference to the AIF measurement discussion above, it is important to select an AIF approach that is most appropriate for the analysis and provides consistency to the study. Thus, another point of consideration in designing a study is the acquisition of the AIF. This will not only affect the manner in which the data is analyzed, but also the actual acquisition protocol. If the study requires a subject-specific AIF, then it will be necessary to either implement blood sampling or to assess the AIF through imaging methods. When utilizing imaging methods to assess the AIF, this will dictate a higher temporal resolution in order to quantify the rapid uptake of the AIF, and it will also require that a blood pool be located in the FOV relative to the ROI.

Intimately linked to the data acquisition methods, is the planned type of data analysis. If a very high spatial resolution is selected (similar to what is done in clinical breast DCE-MR studies), then there are fundamental limitations on the analysis that can be performed. More specifically, if very high spatial resolution data is acquired leading to dynamic data that is sampled quite slowly (say, on the order of 60 s or greater), than only semi-quantitative and qualitative analysis approaches are possible.

Once a balance of spatial, temporal, and SNR demands is struck, it is imperative that the individual lab develop a high level of competence with the selected approach, as opposed to attempting to implement a turn-key technique applied from literature. As mentioned previously, establishing repeatability and reproducibility in a lab is very important, especially prior to analyzing data for changes in parameter values which could potentially be associated with treatment response. 

## 4. Methods of Validating DCE-MRI Analyses

### 4.1. Histology

Before a biomarker is accepted for routine clinical practice, it must be validated such that the measurement reliably predicts clinical outcomes related to therapy [116]. The gold-standard endpoint for most experimental studies is a comparison with histological tissue sections that are processed to report on, for example, cellularity, vascularity, or the expression of a specific protein. Many studies have attempted to correlate *K^trans^*, *v_p_*, and semi-quantitative parameters (e.g., time to peak (*T*_m_)) returned from DCE-MRI analyses with microvessel density (MVD), and thus provide some potential validation (Table 3). MVD measurements are commonly quantified from immunohistochemical tissue sections stained with, for example, CD31, which is a protein expressed on the surface of endothelial cells and mediates cell-cell interactions during angiogenesis [89]. Studies have shown positive and significant correlations between semi-quantitative parameters reflecting changes of MR signal enhancement curve and MVD [117,118]. Hulka *et al.* showed a positive (but weak, *r* = 0.36, *p* < 0.01) correlation between the extraction flow product (*E∙F*, proportional to *K^trans^*) and MVD when benign and malignant tumors were grouped together in the same comparison [119]. However, the authors comment that MVD and *E∙F* were not significantly correlated when each group was compared separately to MVD (benign, *p* = 0.4; malignant, *p* = 0.8). Yao *et al.* also observed a weak positive correlation between *K^trans^* and MVD (*r* = 0.495, *p* = 0.026) [120]. However, several other studies did not observe a correlation between the two parameters [117,121,122]. Conflicting results have also been reported when comparing *v_p_* and MVD. Where one study observed a moderate positive correlation between the two parameters [117], yet another study using two CAs with differing molecular weights observed very weak and negative correlations between *v_p_* and MVD [122].

**Table 3:** Studies comparing DCE-MRI parameters, *K^trans^* and *v_p_*, with histological assessments of microvessel density.

Author, Year (reference)	Tissue of Interest	Histology Technique	MRI Parameter	MVD Correlation ( *r*^2^)	Statistical Significance( *p* value)
Cheng, 2007 [117]	Bladder tissue constructs	IHC-CD31	AUC (60 s)	0.784	0.003
			*K^trans^*	0.4	NS
			*v_p_*	0.696	0.012
Ren, 2008 [118]	Prostate	IHC-CD34	time to peak, *T*_m_	−0.71	<0.007
			Percent enhancement, % *SI*	0.557	<0.007
			Enhancement rate ( *R =* % *SI*/*T*_m_)	0.747	<0.007
Hulka, 1997 [119]	Breast cancer	IHC-factor VIII-related antigen	*E∙F*	0.36	<0.01
Yao, 2008 [120]	Rectal cancer	IHC-CD34	*K^trans^*	0.495	0.026
Haris, 2008 [121]	Brain tuberculomas	IHC-CD34	*K^trans^*	0.231	0.076
Orth, 2007 [122]	Breast cancer xenografts	IHC-CD31	*K^trans^* (Gadomer-17)	0.13	0.659
			*v_b_* (Gadomer-17)	−0.081	0.782
			*K^trans ^*(Magnevist)	0.045	0.874
			*v_b_* (Magnevist)	−0.15	0.594
Reitan, 2010 [123]	Osteosarcoma xenografts	Fluorescently-labeled Dextran	*K^trans^*	0.93	0.04

Cheng *et al.* and Mayr *et al.* state that a lack of correlation between DCE-MRI parameters and histology does not necessarily indicate inaccuracy of the parameter, but perhaps that the complex and continuously changing metabolic demands of tumor angiogenesis is not adequately sampled by histological techniques [117,124]. For example, MVD immunostaining reports only a morphologic index of tumor vasculature and cannot differentiate between functional vessels, whereas *K^trans^* (and *v_p_*) reflects only those vessels with active perfusion. Evidence to support the previous statements is shown in two recent studies where a significant decrease in *K^trans^* after treatment was observed in mouse models of human lung [89] and breast [91] cancers, but the observed decrease in MVD was not significant. 

Depending on the CA or tissue of interest, *K^trans^* might be reflecting more on vessel permeability rather than flow, as previously discussed. Reitan *et al.* used a dorsal window chamber and fluorescently tagged dextran to study the relationship between *K^trans^* from DCE-MRI and vessel permeability. They observed a significant correlation (*r* = 0.93, *p* = 0.04) between DCE-MRI derived *K^trans^* and confocal laser scanning microscopy with fluorescently-labeled Dextran derived extravasation rate, *K_i_* [123]. 

Hemotoxlylin and eosin (H & E) is a histology technique that can potentially be used to validate *v_e_*. Egeland *et al.* observed a significant correlation (*r* = 0.97; *p* = 0.014) between MR-derived and histology-derived *v_e_* measurements across multiple melanoma xenografts [125]. Aref *et al.* also observed good correlation, and did not observe a significant difference (*p* = 0.97) between the two parameters [126]. It should be noted, however, that regions of tumor necrosis were not incorporated into the data analyses of the two studies. Egeland *et al.* commented that the multiple tumor xenografts were well-vascularized and did not develop large necrotic regions [125]. Aref *et al.* used a thresholding technique to include only those *v_e_* voxels that had corresponding “top-five” volume-normalized *K^trans^* voxels [126]. Thus, the significant correlations observed in these studies between MR and histology estimates of *v_e_* existed due to the inclusion of only well-vascularized tumor regions. 

Several groups have developed methods to co-localize histology tissue sections with *in vivo* MRI data. Sinha *et al.* acquired digital images from multiple blockface sections collected using a cryomicrotome that were then registered with both the *in vivo* imaging data and histological sections, thus co-registering MR images with histology [127]. Meyer *et al.* also developed a similar registration technique to co-localize MR and histology data, but with an additional step of acquiring an *ex vivo* high-resolution image of the entire sample to maximize mutual information between the *in vivo* MRI, tissue block photograph, or stained histological slide [128]. Zanzonico designed and implemented a different technique that included a stereotactic template with fiduciary markers for spatial registration of macroscopic *in vivo* imaging data and microscopic images of histological sections [129]. This method requires that the animal be quickly sacrificed immediately following imaging, as well as the placement of angiocatheters into the fiduciary marker holes and through the entire tumor volume. The fiduciary markers and angiocatheter holes are easily located on all images for image registration. These methods are attractive in that they allow for voxel-based comparisons of DCE-MRI parameters (*i.e.K^trans^* and *v_e_*) with their histological counterparts, instead of ROI-based techniques computed from the whole tumor volume or slice. However, it should be noted that registering, and thus co-localizing, histology data that is on the order of ~10 microns with *in vivo* imaging data that is on the order of ~1000 microns is not trivial. Following a strict protocol with a high level of detail is essential during tissue sectioning to ensure proper alignment between the images of extremely different resolutions; see, e.g., the methods developed by Sinha *et al.* [98].

### 4.2. Dynamic Contrast Enhanced Computed Tomography

Dynamic contrast enhanced computed tomography (DCE-CT) is a technique similar to DCE-MRI in that images are collected before, during, and after injection of a contrast agent. In principle, DCE-CT might provide more robust measurements of tumor microvasculature compared to DCE-MRI, as the concentration of CA is directly proportional to the measured Hounsfield units, thereby greatly simplifying the dynamic analysis. However, DCE-CT has some disadvantages that limit clinical utility; in particular, limitations on the quantity of ionizing radiation dose to the subject can lead to compromises in image contrast, volume coverage, and temporal resolution during acquisition [130].

Several groups have performed studies to compare vascular parameters returned from both DCE-MRI and DCE-CT analyses, and thus provide some potential validation for DCE-MRI kinetic parameters. In a study involving bladder cancer, Naish *et al.* analyzed both MR and CT dynamic data with the extended Kety-Tofts compartmental model, and observed good agreement with an intramodality coefficient of variation (CV) of 9% between the MR-derived and CT-derived *K^trans^* [130]. A lower level of agreement was observed with *v_e_* and *v_p _*with CV’s of 53% and 50%, respectively. Korporaal *et al.* quantified *K^trans^* and did not observe significant differences between modality in tumor (Wilcoxon Signed-rank; *p* = 0.18) or healthy tissue (*p* = 0.38) in patients with local prostate cancer [131]. Kierkels *et al.* observed a significant correlation between modalities with *K^trans^* (Kendall’s tau; *τ* = 0.81, *p* < 0.001) in a study of rectal cancer; however, no correlation occurred with *v_e_* (*τ* = −0.35, *p* < 0.2) or *v_p_* (*τ* = 0.23, *p* < 0.4) [132]. The results from these studies suggest that DCE-CT can provide an *in vivo* method to validate MR-derived *K^trans^* values. The lower agreement between quantitative measurements of *v_e_* and *v_p_* suggest that these parameters may need to be interpreted with caution in, for example, a longitudinal study with an anti-angiogenic therapy.

### 4.3. Other Modalities

As noted above, there are certain situations in regions of tissue that can lead to unreliable estimates of *v_e_* as estimated by DCE-MRI. The data on correlation between DCE-MRI and DCE-CT just presented indicated a modest correlation between CT and MRI derived estimates of *v_e_* [130,132,133]. While the reasons for this discrepancy are not entirely clear (though it is possible that it is due to the different molecular weights and/or permeabilities of the different CAs used for each modality), there are other nuclear techniques, both *in vivo* and *ex vivo*, that can potentially be used to validate *v_e_*. These methods are attractive in that they do not rely on assumptions about water dynamics or require measurement of the AIF. These techniques measure radiotracer activities from the tissues of interest and then compare to a reference standard (e.g. measured activity of a blood sample) to quantify radiotracer concentration in the extravascular-extracellular space. Donahue *et al.* used *ex vivo* autoradiography to estimate the cell volume fraction (=1 − *v_e_*) from whole tissue samples, and observed percent differences between modalities of less than 5% and 15% for muscle and tumor, respectively [134]. Skinner *et al.* employed an *in vivo* technique using quantitative dual-isotope single photon emission computed tomography (SPECT) to obtain absolute measurements of *v_e_* on a voxel-by-voxel basis in a rat glioma model, and observed a systematic overestimation of MR-derived estimates of *v_e_* compared to SPECT [93]. The authors discuss several possible explanations for the overestimation of *v_e_*, including effects on MR signal intensity from in-flowing blood and water-exchange between tissue compartments; however, they hypothesize the greatest effect to be due to differences in CA dynamics between well-vascularized and necrotic tumor regions. The Kety-Tofts models will assign very low (non-zero) *K^trans^* values to poorly perfused regions, which will thus overestimate *v_e_* [135]. (We return to this point of CA diffusion and overestimation of *v_e_* from DCE-MRI in Section 6. This SPECT measurement of *v_e_* is insensitive to CA diffusion as time was allowed for radiotracer distribution between injection and imaging.) 

## 5. Relationships to Other Imaging Modalities

### 5.1. Possible Relationships between DCE-MRI and DW-MRI

Self-diffusion or Brownian motion is the microscopic thermally induced behavior of water molecules moving in a random pattern [136]. The rate of self-diffusion in tissues is described by the apparent diffusion coefficient (ADC), which largely depends on the number of semipermeable barriers (e.g., cell membranes), that moving water molecules encounter. Imaging techniques such as diffusion-weighted (DW) MRI have been developed to quantify ADC; in well-controlled situations, variations in ADC have been correlated with cellularity [137]. In the literature, *v_e_* has been shown to correlate with cellular density and the extravascular-extracellular volume fraction from histology [16,126]. Thus, it is natural to compare ADC and *v_e_*. One of the first studies to examine the relationship between *v_e_* and ADC simultaneously was performed in the context of neoadjuvant chemotherapy in breast cancer [138]. In this study, DCE-MRI and DW-MRI were acquired both pre- and post-treatment in order to determine the sensitivity of these two techniques in assessing treatment response. Interestingly, this study found a negative correlation between ADC and *v_e_*. These results seem counterintuitive, as a reduction in cell density due to effective treatment should presumably lead to an increase in both ADC and *v_e_*. Another study examining ADC and *v_e_* in glioblastomas observed no relationship between the two parameters [139]. Furthermore, Arlinghaus *et al.* did not observe any statistical significance between ADC and *v_e_* in patients with breast cancer [140]. The authors hypothesize that current DCE-MRI estimates of *v_e_* (e.g., Kety-Tofts models) incorporate assumptions that might not be valid for data analysis of specific tissues, especially necrotic regions of tumors that are poorly perfused. Necrotic regions will present with inaccurate measurements of *v_e_* and a wide range of ADC values, thus lowering the chance for any statistical significance between the two parameters. (We address this issue in more detail in Section 6.)

### 5.2. Possible Relationships between DCE-MRI and Common PET Tracers

#### 5.2.1. PET Imaging of Hypoxia

A tumor will quickly outgrow its blood supply as it proliferates, resulting in pockets of hypoxia that are heterogeneously spaced throughout the tumor. Hypoxia causes tumor cells to release specific cytokines and growth factors that activate normal stromal and endothelial cells of the tumor microenvironment and initiate angiogenesis [1], thus leading to a possible relationship between DCE-MRI and imaging techniques designed to estimate hypoxia. Positron emission tomography (PET) with specific radiotracers designed to accumulate in regions of low oxygen content have been extensively investigated [141,142,143]; here we focus on one of the leading PET agents for imaging hypoxia, ^18^F-fluoromisonidazole (^18^F-FMISO). ^18^F-FMISO is a radiolabeled nitroimidazole that freely diffuses through the cell membrane, but retention is determined by the local oxygen tension. This relationship between tumor hypoxia and angiogenesis using ^18^F-FMISO and DCE-MRI has been previously investigated in a preclinical model of cancer. Cho *et al*. performed co-registration of MRI and PET images to investigate the spatial correlation between tumor perfusion and hypoxia [144]. They observed a negative correlation between perfusion as assessed by DCE-MRI and hypoxia measured by late ^18^F-FMISO uptake, which in this study was quantified as the linear slope of the dynamic data from the last hour of image acquisition [144]. This observation provides evidence for the hypothesis that poorly perfused tumors are hypoxic [145]. A clinical study performed by Jansen *et al.* also found a moderate negative correlation (Spearman rank; *ρ* = −0.36) between *K^trans^* and ^18^F-FMISO standardized uptake value (SUV, ratio of tissue concentration and injected activity at a certain time after injection normalized to subject weight) in Head and Neck cancer patients with neck nodal metastasis [146]. These findings also support the hypothesis that an inverse relationship exists between tissue perfusion and hypoxia.

#### 5.2.2. PET Imaging of Glycolysis

In addition to inducing angiogenesis, hypoxia has also been implicated in the transcription of the cell-surface glucose transporter GLUT-1 and at least one of the primary enzymes in glycolysis [147]. This leads to another possible relationship between DCE-MRI and imaging techniques of glycolysis. PET imaging of glycolysis is not new; in fact, the radiotracer ^18^F-2'-fluoro-2'-deoxyglucose (^18^F-FDG) is currently a standard-of-care approach for staging metastatic disease in many cancers. ^18^F-FDG is a glucose analogue that is actively taken up in cells via the GLUT-1 and GLUT-3 transporters and phosphorylated by hexokinase; however, once phosphorylated it is not metabolized further in the glycolytic pathway and remains trapped in the cell. The rate of phosphorylation is proportional to the metabolic rate of the cell; thus, cells with high metabolic rates will accumulate more ^18^F-FDG. Metz *et al.* and Partridge *et al.* investigated the spatial relationship of glucose metabolism and microcirculation in non-small cell lung [148] and breast [149] cancers, respectively. Metz *et al.* found a positive but moderate correlation (*r* = 0.52, *p* < 0.05) between AUC from DCE-MRI and ^18^F-FDG SUV. Partridge *et al.* acquired and processed dynamic ^18^F-FDG data, and also found a positive but stronger correlation between AUC and parameters relating to ^18^F-FDG delivery into the tissue (*K*_1_, *r* = 0.61, *p* = 0.019) and metabolism flux constant (*K_i_*, *r* = 0.76, *p* = 0.002). These data suggest that a stronger spatial correlation exists when both imaging modalities are acquired and processed dynamically; thus, it would be worthwhile to investigate the relationship between ^18^F-FDG dynamic data and parameters (e.g., *K^trans^*,*v_e_*, *v_p_*) returned from analyzing DCE-MRI data with a pharmacokinetic model that better reflects tissue contrast agent kinetics. Both DCE-MRI and ^18^F-FDG dynamic analyses have been implemented in many preclinical models of cancer (see, e.g., [89,150]) and patients (e.g., [151,152]) to investigate the efficacy of assessing therapeutic response. A more thorough knowledge of the relationship between these two modalities could improve patient care in both the diagnostic and prognostic setting; to this avail, there are many studies investigating the predictive value of multi-parametric imaging [26,153,154]. 

#### 5.2.3. PET Imaging of Cell Proliferation

**Figure 5:**
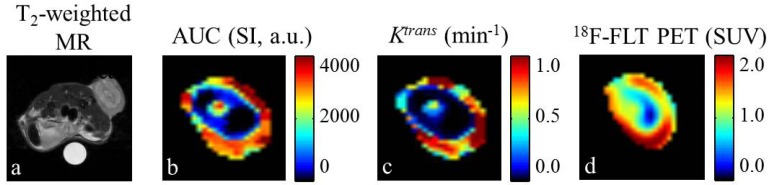
Parametric images resulting from the analysis of DCE-MRI (panels **b**,**c**) and ^18^F-FLT data (panel **d**). A T_2_-weighted MR anatomical shows the entire imaging field of view (panel **a**). Note the spatial correlation between *K^trans^* and SUV, thus one might look to investigate a potential relationship between *K^trans^* and cell proliferation. (Please note that image co-registration between MRI and PET data was not performed in this example.)

Functional tumor vascular structures imaged with DCE-MRI are preceded by endothelial cell proliferation, which again leads to another hypothesis concerning the relationship between DCE-MRI and imaging techniques designed to estimate cell proliferation. A promising PET tracer that reports on cell proliferation is the thymidine analogue 3'-deoxy-3'-^18^F-fluorothymidine (^18^F-FLT). ^18^F-FLT retention is regulated by the cell-cycle dependent thymidine salvage pathway and activity of thymidine kinase 1 (TK1), which is upregulated during the DNA synthesis phase. Thus, ^18^F-FLT PET provides an estimate of cell proliferation. To the best of our knowledge, the relationship between DCE-MRI and ^18^F-FLT PET has not been previously investigated. Figure 5 shows examples of parametric maps generated from images acquired in an ongoing multimodality study at our institution. DCE-MRI data are displayed with parametric maps of AUC at 90 s post injection (panel b), and *K^trans ^* (panel c, quantified via the extended Kety-Tofts model with the population average AIF from Loveless *et al.* [81]). A parametric map of SUV from ^18^F-FLT PET data is shown in panel d. These images reveal a potential spatial correlation between *K^trans^*, AUC, and SUV; thus, one might hypothesize that a direct relationship exists between *K^trans^* and proliferation. The SUV quantifies a snapshot of the activity that exists within the tissue at some time after radiotracer injection, and does not distinguish between the radiotracer collecting in the interstitial space due to nonspecific binding and what accumulates within the cell after TK_1_ phosphorylation. Therefore, the hypothesis that *K^trans^* and cell proliferation are directly related might not be true despite the observed spatial correlation between *K^trans^* and SUV in this particular example. Regardless, this is data from only one mouse and is meant to be illustrative of the types of investigations the cancer imaging community should explore in future multi-modality studies. As many of these parameters are complimentary in nature, it will be important to investigate how their relationships are linked to the underlying biology.

## 6. Limitations

**Figure 6:**
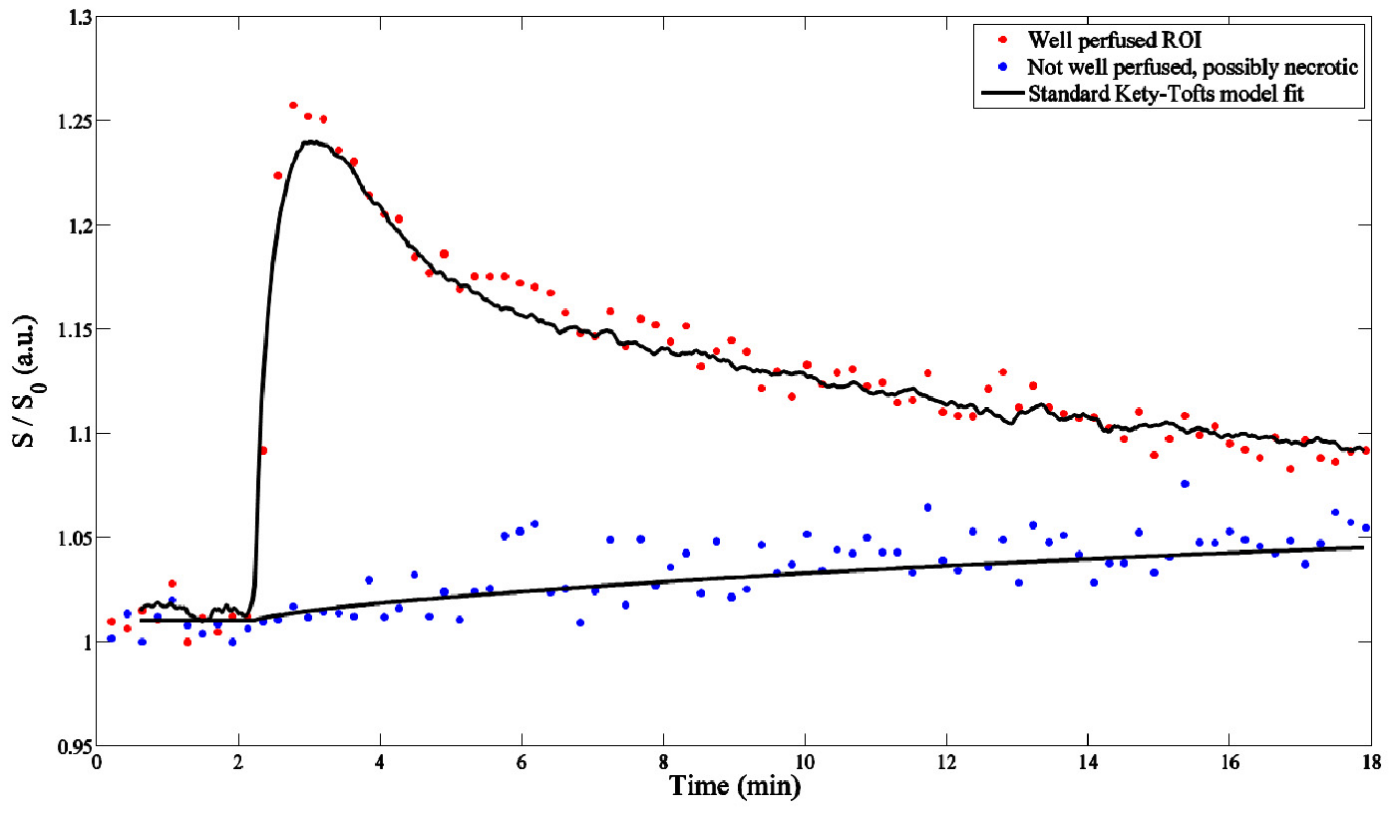
Representative signal intensity curves from DCE-MRI data demonstrating dynamic time courses from a well perfused region and the potential effect of contrast agent diffusion in the extravascular space. Time courses are also shown with a curve fit from standard Kety-Tofts model; *K^trans^* (min^−1^) is 0.92 and <0.001 (almost zero) for the well perfused and necrotic region, respectively, *v_e_* is 0.44 and 1.6 for the well perfused and necrotic region, respectively. The effect of diffusion can be seen by the persistence of the contrast agent in the latter portion of the curves, specifically in the curve labeled as “possibly necrotic”. This diffusion effect causes the model to return *v_e_* values that are unphysiological (*i.e.*, *v_e_* > 1).

In considering quantitative DCE-MRI, potential limitations exist in the models utilized in the analysis. One example is the effect of diffusion on the tissue system. The standard and extended Kety-Tofts models account for active delivery of CA via the vasculature as well as exchange of the CA between the vascular space and the extravascular-extracellular space [155,156,157]; however, in general, these models neglect passive diffusion of CA that may occur within the tissue. This issue is of specific concern in areas where well-perfused tissue is in proximity to poorly-perfused tissue, as is often the case in pathologic tissue such as tumors [158]. If diffusion plays a role in DCE-MRI data, then the current quantitative models may assign parameters that are inaccurate. Though this potential effect of diffusion has been recognized, the literature regarding the effect is limited. Pellerin *et al.* acknowledged the effect of diffusion and used a finite difference model to study the effect [135]. The authors developed a diffusion-perfusion (DP) model that incorporated diffusion coefficients derived from the ADC values to assess inter-voxel diffusion. Optimization of the model allowed for voxel-wise calculation of *K^trans^* and *v_e_*, which showed a quantifiable improvement in parameterization versus the standard model in simulation cases and in a murine model of cancer. In another interrogation of diffusion, Jia *et al.* calculated a contrast agent diffusion coefficient (CDC) in colorectal liver metastases [159]. The authors quantified the CDC by fitting the gradient of the signal intensity curves within the tumor ROI to a monoexponential decay. This resulted in a decay factor that related to the CDC and described the heterogeneity of the tissue. In addition to quantifying diffusion, the authors also provided visual confirmation of the effect of diffusion within the various layers of the lesion. This was achieved by applying an onion-peeling algorithm to the data, which extracted pixel-deep layers of the lesion. By visualizing the SI curves of each layer, the effect of diffusion was apparent from the shape of the curves, specifically during the extravascular phase. An example of the various curve shapes typically seen in DCE-MRI is shown in Figure 6; in these images, the potential effect of diffusion is visible as the persistence of the signal in the latter portion of the curves.

Another potential limitation of the current quantitative models is the assumption of a well-mixed extravascular-extracellular space. Standard modeling assumes a linear dependence of the relaxation rate on the CA concentration (Equation 1). However, studies have shown that the exchange between the intracellular and extracellular space may not be rapid enough to ensure a homogenous system thus violating the assumption of linear CA dependence; to this avail, authors have considered the extravascular-intracellular space as a secondary water pool distinct from the extravascular-extracellular water [160,161,162]. The traditional standard and extended Kety-Tofts models assume that the extravascular space is a homogeneous solution and that the system remains in what is called the “fast exchange limit” (FXL) with respect to the water exchange between the extravascular extracellular space and the extravascular intracellular space. But in most tissues, most water is intracellular, and because the common Gd chelates cannot access the intracellular space, water exchange between the extravascular extracellular space and the extravascular intracellular space may need to be explicitly incorporated into analytic models [161,162,163,164]. Similar comments apply to water exchange between the intravascular and extravascular spaces when using an intravascular agent [165]. In these cases, longitudinal relaxation may not be well described by a single exponential [160,166,167]. The effects of slower water exchange may be incorporated into analyses of the DCE-MRI data. As one might guess, the estimated pharmacokinetic parameters may differ significantly [35,161,162,163,164,168] depending on the model that was used to extract them, and these differences may have a significant effect on establishing if, for example, a tumor is responding to treatment. However, there is some disagreement as to whether this formalism is truly justified in a standard DCE-MRI study and it is therefore an active area of investigation [37,38].

These results, along with those presented for the effect of diffusion, make it clear that the quantitative models generally used for DCE-MRI analysis are most likely simplifications of the true physiologic behavior of the tissue, a point that needs to be considered when drawing conclusions from quantitative parameters. 

## 7. Summary

DCE-MRI has the potential to serve as a biomarker of disease progression and response to treatment. Preclinical applications of DCE-MRI play a pivotal (and central) role in the development and advancement of the technique. In this review, we have explored the practical considerations necessary to develop a preclinical study, including background information on DCE-MRI, the necessary acquisition and analysis components, and the points that should be considered before executing a study. Additionally, we have provided a discussion of the methods of validating DCE-MRI, as well as a discussion of the correlation of DCE parameters to other imaging modalities. It is our hope that this review will provide encouragement and useful guidance to incorporate DCE-MRI into preclinical studies of anti-cancer therapies.

## References

[1] Weis S.M., Cheresh D.A. (2011). Tumor angiogenesis: Molecular pathways and therapeutic targets. Nat. Med..

[2] Ferrara N., Gerber H., LeCouter J. (2003). The biology of vegf and its receptors. Nat. Med..

[3] Young R., Reed M. (2012). Anti-angiogenic therapy: Concept to clinic. Microcirculation.

[4] Vredenburgh J.J., Desjardins A., Herndon J.E., Dowell J.M., Reardon D.A., Quinn J.A., Rich J.N., Sathornsumetee S., Gururangan S., Wagner M. (2007). Phase ii trial of bevacizumab and irinotecan in recurrent malignant glioma. Clin.Cancer Res..

[5] Hurwitz H., Fehrenbacher L., Novotny W., Cartwright T., Hainsworth J., Heim W., Berlin J., Baron A., Griffing S., Holmgren E. (2004). Bevacizumab plus irinotecan, fluorouracil, and leucovorin for metastatic colorectal cancer. N. Engl. J. Med..

[6] Miller K., Wang M., Gralow J., Dickler M., Cobleigh M., Perez E.A., Shenkier T., Cella D., Davidson N.E. (2007). Paclitaxel plus bevacizumab *versus* paclitaxel alone for metastatic breast cancer. N. Engl. J. Med..

[7] Saltz L.B., Clarke S., Diaz-Rubio E., Scheithauer W., Figer A., Wong R., Koski S., Lichinitser M., Yang T.S., Rivera F. (2008). Bevacizumab in combination with oxaliplatin-based chemotherapy as first-line therapy in metastatic colorectal cancer: A randomized phase iii study. J. Clin. Oncol..

[8] Friedman H.S., Prados M.D., Wen P.Y., Mikkelsen T., Schiff D., Abrey L.E., Yung W.K., Paleologos N., Nicholas M.K., Jensen R. (2009). Bevacizumab alone and in combination with irinotecan in recurrent glioblastoma. J. Clin. Oncol..

[9] Miles D.W., Chan A., Dirix L.Y., Cortes J., Pivot X., Tomczak P., Delozier T., Sohn J.H., Provencher L., Puglisi F. (2010). Phase iii study of bevacizumab plus docetaxel compared with placebo plus docetaxel for the first-line treatment of human epidermal growth factor receptor 2-negative metastatic breast cancer. J. Clin. Oncol..

[10] Robert N.J., Dieras V., Glaspy J., Brufsky A.M., Bondarenko I., Lipatov O.N., Perez E.A., Yardley D.A., Chan S.Y., Zhou X. (2011). Ribbon-1: Randomized, double-blind, placebo-controlled, phase iii trial of chemotherapy with or without bevacizumab for first-line treatment of human epidermal growth factor receptor 2-negative, locally recurrent or metastatic breast cancer. J. Clin. Oncol..

[11] Demetri G.D., van Oosterom A.T., Garrett C.R., Blackstein M.E., Shah M.H., Verweij J., McArthur G., Judson I.R., Heinrich M.C., Morgan J.A. (2006). Efficacy and safety of sunitinib in patients with advanced gastrointestinal stromal tumour after failure of imatinib: A randomised controlled trial. Lancet.

[12] Escudier B., Eisen T., Stadler W.M., Szczylik C., Oudard S., Siebels M., Negrier S., Chevreau C., Solska E., Desai A.A. (2007). Sorafenib in advanced clear-cell renal-cell carcinoma. N. Engl. J. Med..

[13] Llovet J.M., Ricci S., Mazzaferro V., Hilgard P., Gane E., Blanc J.F., de Oliveira A.C., Santoro A., Raoul J.L., Forner A. (2008). Sorafenib in advanced hepatocellular carcinoma. N. Engl. J. Med..

[14] Motzer R.J., Hutson T.E., Tomczak P., Michaelson M.D., Bukowski R.M., Oudard S., Negrier S., Szczylik C., Pili R., Bjarnason G.A. (2009). Overall survival and updated results for sunitinib compared with interferon alfa in patients with metastatic renal cell carcinoma. J. Clin. Oncol..

[15] Motzer R.J., Hutson T.E., Tomczak P., Michaelson M.D., Bukowski R.M., Rixe O., Oudard S., Negrier S., Szczylik C., Kim S.T. (2007). Sunitinib *versus* interferon alfa in metastatic renal-cell carcinoma. N. Engl. J. Med..

[16] Tofts P.S., Brix G., Buckley D.L., Evelhoch J.L., Henderson E., Knopp M.V., Larsson H.B.W., Lee T.Y., Mayr N.A., Parker G.J.M. (1999). Estimating kinetic parameters from dynamic contrast-enhanced t1-weighted mri of a diffusable tracer: Standardized quantities and symbols. J. Magn. Reson. Imag..

[17] Yankeelov T.E., Gore J.C. (2007). Dynamic contrast enhanced magnetic resonance imaging in oncology: Theory, data, acquisition, analysis, and examples. Curr. Med. Imag. Rev..

[18] Lee L., Sharma S., Morgan B., Allegrini P., Schnell C., Brueggen J., Cozens R., Horsfield M., Guenther C., Steward W. (2006). Biomarkers for assessment of pharmacologic activity for a vascular endothelial growth factor (vegf) receptor inhibitor, ptk787/zk 222584 (ptk/zk): Translation of biological activity in a mouse melanoma metastasis model to phase i studies in patients with advanced colorectal cancer with liver metastases. Cancer Chemother. Pharmacol..

[19] Beauregard D.A., Thelwall P.E., Chaplin D.J., Hill S.A., Adams G.E., Brindle K.M. (1998). Magnetic resonance imaging and spectroscopy of combretastatin a4 prodrug-induced disruption of tumour perfusion and energetic status. Br. J. Cancer..

[20] Maxwell R.J., Wilson J., Prise V.E., Vojnovic B., Rustin G.J., Lodge M.A., Tozer G.M. (2002). Evaluation of the anti-vascular effects of combretastatin in rodent tumours by dynamic contrast enhanced mri. NMR Biomed..

[21] Robinson S.P., McIntyre D.J.O., Checkley D., Tessier J.J., Howe F.A., Griffiths J.R., Ashton S.E., Ryan A.J., Blakey D.C., Waterton J.C. (2003). Tumour dose response to the antivascular agent zd6126 assessed by magnetic resonance imaging. Br. J. Canc..

[22] Chang Y.C., Yu C.J., Chen C.M., Hu F.C., Hsu H.H., Tseng W.Y.I., Ting-Fang Shih T., Yang P.C., Chih-Hsin Yang J. (2012). Dynamic contrast-enhanced mri in advanced nonsmall-cell lung cancer patients treated with first-line bevacizumab, gemcitabine, and cisplatin. J. Magn. Reson. Imag..

[23] Hoff B.A., Bhojani M.S., Rudge J., Chenevert T.L., Meyer C.R., Galbán S., Johnson T.D., Leopold J.S., Rehemtulla A., Ross B.D. (2012). Dce and dw-mri monitoring of vascular disruption following vegf-trap treatment of a rat glioma model. NMR Biomed..

[24] Hsu C.Y., Shen Y.C., Yu C.W., Hsu C., Hu F.C., Hsu C.H., Chen B.B., Wei S.Y., Cheng A.L., Shih T.T.F. (2011). Dynamic contrast-enhanced magnetic resonance imaging biomarkers predict survival and response in hepatocellular carcinoma patients treated with sorafenib and metronomic tegafur/uracil. J. Hepatol..

[25] Yopp A., Schwartz L., Kemeny N., Gultekin D., Gönen M., Bamboat Z., Shia J., Haviland D., D’Angelica M., Fong Y. (2011). Antiangiogenic therapy for primary liver cancer: Correlation of changes in dynamic contrast-enhanced magnetic resonance imaging with tissue hypoxia markers and clinical response. Ann. Surg. Oncol..

[26] Lemasson B., Christen T., Tizon X., Farion R., Fondraz N., Provent P., Segebarth C., Barbier E.L., Genne P., Duchamp O. (2011). Assessment of multiparametric mri in a human glioma model to monitor cytotoxic and anti-angiogenic drug effects. NMR Biomed..

[27] Heiss W.D., Raab P., Lanfermann H. (2011). Multimodality assessment of brain tumors and tumor recurrence. J. Nucl. Med..

[28] Jacobs M.A., Ouwerkerk R., Wolff A.C., Gabrielson E., Warzecha H., Jeter S., Bluemke D.A., Wahl R., Stearns V. (2011). Monitoring of neoadjuvant chemotherapy using multiparametric, (2)(3)na sodium mr, and multimodality (pet/ct/mri) imaging in locally advanced breast cancer. Breast. Cancer. Res. Treat..

[29] De Bruyne S., van Damme N., Smeets P., Ferdinande L., Ceelen W., Mertens J., van de Wiele C., Troisi R., Libbrecht L., Laurent S. (2012). Value of dce-mri and fdg-pet/ct in the prediction of response to preoperative chemotherapy with bevacizumab for colorectal liver metastases. Br. J. Canc..

[30] Viel T., Talasila K.M., Monfared P., Wang J., Jikeli J.F., Waerzeggers Y., Neumaier B., Backes H., Brekka N., Thorsen F. (2012). Analysis of the growth dynamics of angiogenesis-dependent and angiogenesis-independent experimental glioblastomas by multimodal small-animal pet and mri. J. Nucl. Med..

[31] Jansen J.F.A., Schöder H., Lee N.Y., Stambuk H.E., Wang Y., Fury M.G., Patel S.G., Pfister D.G., Shah J.P., Koutcher J.A. (2012). Tumor metabolism and perfusion in head and neck squamous cell carcinoma: Pretreatment multimodality imaging with 1 h magnetic resonance spectroscopy, dynamic contrast-enhanced mri, and [18f] fdg-pet. Int.J. Radiat. Oncol. Biol. Phys..

[32] Calamante F. (2010). Perfusion mri using dynamic-susceptibility contrast mri: Quantification issues in patient studies. Top Magn. Reson. Imag..

[33] Stanisz G.J., Henkelman R.M. (2000). Gd-dtpa relaxivity depends on macromolecular content. Magn. Reson. Med..

[34] Landis C.S., Li X., Telang F.W., Molina P.E., Palyka I., Vetek G., Springer C.S. (1999). Equilibrium transcytolemmal water-exchange kinetics in skeletal muscle *in vivo*. Magn. Reson. Med..

[35] Li X., Priest R.A., Woodward W.J., Tagge I.J., Siddiqui F., Huang W., Rooney W.D., Beer T.M., Garzotto M.G., Springer C.S. (2012). Feasibility of shutter-speed dce-mri for improved prostate cancer detection. Magn. Reson. Med..

[36] Li X., Priest R.A., Woodward W.J., Siddiqui F., Beer T.M., Garzotto M.G., Rooney W.D., Springer C.S. (2012). Cell membrane water exchange effects in prostate dce-mri. J. Magn. Reson..

[37] Bains L.J., McGrath D.M., Naish J.H., Cheung S., Watson Y., Taylor M.B., Logue J.P., Parker G.J., Waterton J.C., Buckley D.L. (2010). Tracer kinetic analysis of dynamic contrast-enhanced mri and ct bladder cancer data: A preliminary comparison to assess the magnitude of water exchange effects. Magn. Reson. Med..

[38] Buckley D.L., Kershaw L.E., Stanisz G.J. (2008). Cellular-interstitial water exchange and its effect on the determination of contrast agent concentration *in vivo*: Dynamic contrast-enhanced mri of human internal obturator muscle. Magn. Reson. Med..

[39] Caravan P. (2006). Strategies for increasing the sensitivity of gadolinium based mri contrast agents. Chem. Soc. Rev..

[40] Weinmann H.J., Brasch R.C., Press W.R., Wesbey G.E. (1984). Characteristics of gadolinium-dtpa complex: A potential nmr contrast agent. AJR Am. J. Roentgenol..

[41] Kaewlai R., Abujudeh H. (2012). Nephrogenic systemic fibrosis. Am. J. Roentgenol..

[42] Wermuth P.J., Jimenez S.A. (2012). Gadolinium compounds signaling through tlr 4 and tlr 7 in normal human macrophages: Establishment of a proinflammatory phenotype and implications for the pathogenesis of nephrogenic systemic fibrosis. J. Immunol..

[43] Wiesinger B., Kehlbach R., Hemsen J., Bebin J., Bantleon R., Schwenzer N., Spira D., Claussen C.D., Wiskirchen J. (2011). Effects of magnetic resonance imaging contrast agents on human umbilical vein endothelial cells and evaluation of magnetic resonance imaging contrast media-triggered transforming growth factor-beta induction in dermal fibroblasts (hsf) as a model for nephrogenic systemic fibrosis. Invest. Radiol..

[44] Del Galdo F., Wermuth P.J., Addya S., Fortina P., Jimenez S.A. (2010). Nfkappab activation and stimulation of chemokine production in normal human macrophages by the gadolinium-based magnetic resonance contrast agent omniscan: Possible role in the pathogenesis of nephrogenic systemic fibrosis. Ann. Rheum. Dis..

[45] Steger-Hartmann T., Raschke M., Riefke B., Pietsch H., Sieber M.A., Walter J. (2009). The involvement of pro-inflammatory cytokines in nephrogenic systemic fibrosis-A mechanistic hypothesis based on preclinical results from a rat model treated with gadodiamide. Exp. Toxicol. Pathol..

[46] Sieber M.A., Pietsch H., Walter J., Haider W., Frenzel T., Weinmann H.J. (2008). A preclinical study to investigate the development of nephrogenic systemic fibrosis: A possible role for gadolinium-based contrast media. Invest. Radiol..

[47] Rohrer M., Bauer H., Mintorovitch J., Requardt M., Weinmann H.J. (2005). Comparison of magnetic properties of mri contrast media solutions at different magnetic field strengths. Invest. Radiol..

[48] Yang C.T., Chuang K.H. (2012). Gd(iii) chelates for mri contrast agents: From high relaxivity to "smart", from blood pool to blood-brain barrier permeable. Med.Chem.Comm..

[49] Pathak A.P., Penet M.F., Bhujwalla Z.M., Renata P. (2010). MR molecular imaging of tumor vasculature and vascular targets. Advances in genetics.

[50] Barrett T., Kobayashi H., Brechbiel M., Choyke P.L. (2006). Macromolecular mri contrast agents for imaging tumor angiogenesis. Eur. J. Radiol..

[51] Kiessling F., Morgenstern B., Zhang C. (2007). Contrast agents and applications to assess tumor angiogenesis *in vivo* by magnetic resonance imaging. Curr. Med. Chem..

[52] Evelhoch J.L. (1999). Key factors in the acquisition of contrast kinetic data for oncology. J. Magn. Reson. Imag..

[53] McIntyre D.J., Robinson S.P., Howe F.A., Griffiths J.R., Ryan A.J., Blakey D.C., Peers I.S., Waterton J.C. (2004). Single dose of the antivascular agent, zd6126 (n-acetylcolchinol-o-phosphate), reduces perfusion for at least 96 hours in the gh3 prolactinoma rat tumor model. Neoplasia.

[54] Galbraith S.M., Maxwell R.J., Lodge M.A., Tozer G.M., Wilson J., Taylor N.J., Stirling J.J., Sena L., Padhani A.R., Rustin G.J. (2003). Combretastatin a4 phosphate has tumor antivascular activity in rat and man as demonstrated by dynamic magnetic resonance imaging. J. Clin. Oncol..

[55] Medved M., Karczmar G., Yang C., Dignam J., Gajewski T., Kindler H., Vokes E., Maceneany P., Mitchel M., Stadler W. (2004). Semiquantitative analysis of dynamic contrast enhanced mri in cancer patients: Variability and changes in tumor tissue over time. J. Magn. Reson. Imag..

[56] Mross K., Drevs J., Muller M., Medinger M., Marme D., Hennig J., Morgan B., Lebwohl D., Masson E., Ho Y.Y. (2005). Phase i clinical and pharmacokinetic study of ptk/zk, a multiple vegf receptor inhibitor, in patients with liver metastases from solid tumours. Eur. J. Canc..

[57] Hillman G.G., Singh-Gupta V., Zhang H., Al-Bashir A.K., Katkuri Y., Li M., Yunker C.K., Patel A.D., Abrams J., Haacke E.M. (2009). Dynamic contrast-enhanced magnetic resonance imaging of vascular changes induced by sunitinib in papillary renal cell carcinoma xenograft tumors. Neoplasia.

[58] Marzola P., Degrassi A., Calderan L., Farace P., Nicolato E., Crescimanno C., Sandri M., Giusti A., Pesenti E., Terron A. (2005). Early antiangiogenic activity of su11248 evaluated *in vivo* by dynamic contrast-enhanced magnetic resonance imaging in an experimental model of colon carcinoma. Clin. Canc. Res..

[59] Tang J.S., Choy G., Bernardo M., Thomasson D., Libutti S.K., Choyke P.L. (2006). Dynamic contrast-enhanced magnetic resonance imaging in the assessment of early response to tumor necrosis factor alpha in a colon carcinoma model. Investig. Radiol..

[60] Checkley D., Tessier J.J., Kendrew J., Waterton J.C., Wedge S.R. (2003). Use of dynamic contrast-enhanced mri to evaluate acute treatment with zd6474, a vegf signalling inhibitor, in pc-3 prostate tumours. Br. J. Cancer.

[61] Kuhl C., Mielcareck P., Klaschik S., Leutner C., Wardelmann E., Gieseke J., Schild H. (1999). Dynamic breast mr imaging: Are signal intensity time course data useful for differential diagnosis of enhancing lesions. Radiology.

[62] Fischer U., Kopka L., Grabbe E. (1999). Breast carcinoma: Effect of preoperative contrast-enhanced mr imaging on the therapuetic approach. Radiology.

[63] Buadu L., Murakami J., Murayama S., Hashiguchi N., Sakai S., Masuda K., Toyoshima S., Kuroki S., Ohno S. (1996). Breast lesions: Correlation of contrast medium enhancement patterns on mr images with histopathologic findings and tumor angiogenesis. Radiology.

[64] Ei Khouli R.H., Jacobs M.A., Mezban S.D., Huang P., Kamel I.R., Macura K.J., Bluemke D.A. (2010). Diffusion-weighted imaging improves the diagnostic accuracy of conventional 3.0-t breast mr imaging. Radiology.

[65] Yabuuchi H., Matsuo Y., Okafuji T., Kamitani T., Soeda H., Setoguchi T., Sakai S., Hatakenaka M., Kubo M., Sadanaga N. (2008). Enhanced mass on contrast-enhanced breast mr imaging: Lesion characterization using combination of dynamic contrast-enhanced and diffusion-weighted mr images. J. Magn. Reson.Imag..

[66] Qayyum A., Birdwell R.L., Daniel B.L., Nowels K.W., Jeffrey S.S., Agoston T.A., Herfkens R.J. (2002). Mr imaging features of infiltrating lobular carcinoma of the breast: Histopathologic correlation. Am. J. Roentgenol..

[67] Weinstein S.P., Orel S.G., Heller R., Reynolds C., Czerniecki B., Solin L.J., Schnall M. (2001). Mr imaging of the breast in patients with invasive lobular carcinoma. Am. J. Roentgenol..

[68] Orel S.G., Schnall M.D., LiVolsi V.A., Troupin R.H. (1994). Suspicious breast lesions: Mr imaging with radiologic-pathologic correlation. Radiology.

[69] Buadu L.D., Murakami J., Murayama S., Hashiguchi N., Sakai S., Toyoshima S., Masuda K., Kuroki S., Ohno S. (1997). Patterns of peripheral enhancement in breast masses: Correlation of findings on contrast medium enhanced mri with histologic features and tumor angiogenesis. J. Comput. Assist. Tomo..

[70] Galbraith S.M., Lodge M.A., Taylor N.J., Rustin G.J.S., Bentzen S., Stirling J.J., Padhani A.R. (2002). Reproducibility of dynamic contrast-enhanced mri in human muscle and tumours: Comparison of quantitative and semi-quantitative analysis. NMR Biomed..

[71] Kety S.S. (1951). The theory and applications of the exchange of inert gas at the lungs and tissues. Pharmacol. Rev..

[72] Shames D.M., Kuwatsuru R., Vexler V., Mühler A., Brasch R.C. (1993). Measurement of capillary permeability to macromolecules by dynamic magnetic resonance imaging: A quantitative noninvasive technique. Magn. Reson. Med..

[73] Faranesh A.Z., Kraitchman D.L., McVeigh E.R. (2006). Measurement of kinetic parameters in skeletal muscle by magnetic resonance imaging with an intravascular agent. Magn. Reson. Med..

[74] Daldrup H., Shames D., Wendland M., Okuhata Y., Link T., Rosenau W., Lu Y., Brasch R. (1998). Correlation of dynamic contrast-enhanced mr imaging with histologic tumor grade: Comparison of macromolecular and small-molecular contrast media. Am. J. Roentgenol..

[75] Bradley D.P., Tessier J.L., Checkley D., Kuribayashi H., Waterton J.C., Kendrew J., Wedge S.R. (2008). Effects of azd2171 and vandetanib (zd6474, zactima) on haemodynamic variables in an sw620 human colon tumour model: An investigation using dynamic contrast-enhanced mri and the rapid clearance blood pool contrast agent, p792 (gadomelitol). NMR Biomed..

[76] Wedge S.R., Kendrew J., Hennequin L.F., Valentine P.J., Barry S.T., Brave S.R., Smith N.R., James N.H., Dukes M., Curwen J.O. (2005). Azd2171: A highly potent, orally bioavailable, vascular endothelial growth factor receptor-2 tyrosine kinase inhibitor for the treatment of cancer. Canc. Res..

[77] Luo Y., Jiang F., Cole T., Hradil V., Reuter D., Chakravartty A., Albert D., Davidsen S., Cox B., McKeegan E. (2012). A novel multi-targeted tyrosine kinase inhibitor, linifanib (abt-869), produces functional and structural changes in tumor vasculature in an orthotopic rat glioma model. Cancer Chemotherapy and Pharmacology.

[78] Nielsen T., Murata R., Maxwell R.J., Stødkilde-Jørgensen H., Østergaard L., Horsman M.R. (2008). Preclinical studies to predict efficacy of vascular changes induced by combretastatin a-4 disodium phosphate in patients. International Journal of Radiation Oncology*Biology*Physics.

[79] Kingsley P.B. (1999). Methods of measuring spin-lattice (t1) relaxation times: An annotated bibliography. Concepts Magn. Reson..

[80] Guilfoyle D.N., Dyakin V.V., O’Shea J., Pell G.S., Helpern J.A. (2003). Quantitative measurements of proton spin-lattice (t1) and spin-spin (t2) relaxation times in the mouse brain at 7.0 t. Magn. Reson. Med..

[81] Loveless M.E., Halliday J., Liess C., Xu L., Dortch R.D., Whisenant J., Waterton J.C., Gore J.C., Yankeelov T.E. (2012). A quantitative comparison of the influence of individual versus population-derived vascular input functions on dynamic contrast enhanced-mri in small animals. Magn. Reson. Med..

[82] Haase A., Frahm J., Matthaei D., Hanicke W., Merboldt K.D. (1986). Flash imaging. Rapid nmr imaging using low flip-angle pulses. J. Magn. Reson..

[83] Haacke M., Brown R., Thompson M., Venkatesan R. (1999). Magnetic Resonance Imaging: Physical Principles and Sequence Design.

[84] Loveless M.E., Whisenant J.G., Wilson K., Lyshchik A., Sinha T.K., Gore J.C., Yankeelov T.E. (2009). Coregistration of ultrasonography and magnetic resonance imaging with a preliminary investigation of the spatial colocalization of vascular endothelial growth factor receptor 2 expression and tumor perfusion in a murine tumor model. Mol. Imag..

[85] Yankeelov T.E., Niermann K.J., Huamani J., Kim D.W., Quarles C.C., Fleischer A.C., Hallahan D.E., Price R.R., Gore J.C. (2006). Correlation between estimates of tumor perfusion from microbubble contrast-enhanced sonography and dynamic contrast-enhanced magnetic resonance imaging. J. Ultrasound. Med..

[86] Yankeelov T.E., Gore J.C. (2009). Dynamic contrast enhanced magnetic resonance imaging in oncology: Theory, data acquisition, analysis, and examples. Curr. Med. Imag. Rev..

[87] Hornak J.P., Szumowski J., Bryant R.G. (1988). Magnetic field mapping. Magn. Reson. Med..

[88] Yarnykh V.L. (2007). Actual flip-angle imaging in the pulsed steady state: A method for rapid three-dimensional mapping of the transmitted radiofrequency field. Magn. Reson. Med..

[89] Loveless M.E., Lawson D., Collins M., Nadella M.V., Reimer C., Huszar D., Halliday J., Waterton J.C., Gore J.C., Yankeelov T.E. (2012). Comparisons of the efficacy of a jak 1/2 inhibitor (azd 1480) with a vegf signaling inhibitor (cediranib) and sham treatments in mouse tumors using dce-mri, dw-mri, and histology. Neoplasia.

[90] Benjaminsen I.C., Graff B.A., Brurberg K.G., Rofstad E.K. (2004). Assessment of tumor blood perfusion by high-resolution dynamic contrast-enhanced mri: A preclinical study of human melanoma xenografts. Magn. Reson. Med..

[91] Kim H., Folks K., Guo L., Stockard C., Fineberg N., Grizzle W., George J., Buchsbaum D., Morgan D., Zinn K. (2011). Dce-mri detects early vascular response in breast tumor xenografts following anti-dr5 therapy. Mol. Imag. Biol..

[92] Li X., Rooney W.D., Várallyay C.G., Gahramanov S., Muldoon L.L., Goodman J.A., Tagge I.J., Selzer A.H., Pike M.M., Neuwelt E.A. (2010). Dynamic-contrast-enhanced-mri with extravasating contrast reagent: Rat cerebral glioma blood volume determination. J. Magn. Reson..

[93] Skinner J., Yankeelov T.E., Peterson T., Does M. (2012). Comparison of dynamic contrast enhanced mri and quantitative spect in a rat glioma model. Contrast Media Mol. Imag..

[94] Kim J.H., Im G.H., Yang J., Choi D., Lee W.J., Lee J.H. (2012). Quantitative dynamic contrast-enhanced mri for mouse models using automatic detection of the arterial input function. NMR Biomed..

[95] Yankeelov T.E., Luci J.J., Lepage M., Li R., Debusk L., Lin P.C., Price R.R., Gore J.C. (2005). Quantitative pharmacokinetic analysis of dce-mri data without an arterial input function: A reference region model. Magn. Reson. Imag..

[96] Fritz-Hansen T., Rostrup E., Larsson H.B., Sondergaard L., Ring P., Henriksen O. (1996). Measurement of the arterial concentration of gd-dtpa using mri: A step toward quantitative perfusion imaging. Magn. Reson. Med..

[97] Van Osch M.J.P., Vonken E.-j.P.A., Viergever M.A., van der Grond J., Bakker C.J.G. (2003). Measuring the arterial input function with gradient echo sequences. Magn. Reson. Med..

[98] Port R.E., Knopp M.V., Hoffmann U., Milker-Zabel S., Brix G. (1999). Multicompartment analysis of gadolinium chelate kinetics: Blood-tissue exchange in mammary tumors as monitored by dynamic mr imaging. J. Magn. Reson. Med..

[99] Port R.E., Knopp M.V., Brix G. (2001). Dynamic contrast-enhanced mri using gd-dtpa: Interindividual variability of the arterial input function and consequences for the assessment of kinetics in tumors. Magn. Reson. Med..

[100] Parker G.J., Barker G.J., Tofts P.S. (2001). Accurate multislice gradient echo t (1) measurement in the presence of non-ideal rf pulse shape and rf field nonuniformity. Magn. Reson. Med..

[101] Parker G.J.M., Roberts C., Macdonald A., Buonaccorsi G.A., Cheung S., Buckley D.L., Jackson A., Watson Y., Davies K., Jayson G.C. (2006). Experimentally-derived functional form for a population-averaged high-temporal-resolution arterial input function for dynamic contrast-enhanced mri. Magn. Reson. Med..

[102] McGrath D.M., Bradley D.P., Tessier J.L., Lacey T., Taylor C.J., Parker G.J.M. (2009). Comparison of model-based arterial input functions for dynamic contrast-enhanced mri in tumor bearing rats. Magn. Reson. Med..

[103] Li X., Welch E.B., Arlinghaus L.R., Chakravarthy A.B., Xu L., Farley J., Loveless M.E., Mayer I.A., Kelley M.C., Meszoely I.M. (2011). A novel aif tracking method and comparison of dce-mri parameters using individual and population-based aifs in human breast cancer. Phys. Med. Biol..

[104] Pickup S., Zhou R., Glickson J. (2003). Mri estimation of the arterial input function in mice. Acad. Radiol..

[105] Kovar D.A., Lewis M., Karczmar G.S. (1998). A new method for imaging perfusion and contrast extraction fraction: Input functions derived from reference tissues. J. Magn. Reson. Imag..

[106] Yang C., Karczmar G.S., Medved M., Stadler W.M. (2004). Estimating the arterial input function using two reference tissues in dynamic contrast-enhanced mri studies: Fundamental concepts and simulations. Magn. Reson. Med..

[107] Heisen M., Fan X., Buurman J., van Riel V., Karczmar G., ter Haar Romeny B. (2010). The use of a reference tissue arterial input function with low-temporal-resolution dce-mri data. Phys. Med. Biol..

[108] Yankeelov T.E., Cron G.O., Addison C.L., Wallace J.C., Wilkins R.C., Pappas B.A., Santyr G.E., Gore J.C. (2007). Comparison of a reference region model with direct measurement of an aif in the analysis of dce-mri data. Magn. Reson. Med..

[109] Yankeelov T.E., DeBusk L.M., Billheimer D.D., Luci J.J., Lin P.C., Price R.R., Gore J.C. (2006). Repeatability of a reference region model for analysis of murine dce-mri data at 7 t. J. Magn. Reson. Imag..

[110] Walker-Samuel S., Parker C.C., Leach M.O., Collins D.J. (2007). Reproducibility of reference tissue quantification of dynamic contrast-enhanced data: Comparison with a fixed vascular input function. Phys. Med. Biol..

[111] Padhani A.R., Hayes C., Landau S., Leach M.O. (2001). Reproducibility of quantitative dynamic mri or normal human tissues. NMR Biomed..

[112] Morgan B., Utting J.F., Higginson A., Thomas A.L., Steward W.P., Horsfield M.A. (2006). A simple, reproducible method for monitoring the treatment of tumours using dynamic contrast-enhanced mr imaging. Br. J. Cancer.

[113] Barnes S., Whisenant J., Loveless M., Ayers G., Yankeelov T. (2012). Assessing the reproducibility of dynamic contrast enhanced magnetic resonance imaging in a murine model of breast cancer. Magn. Reson. Med..

[114] Fueger B.J., Czernin J., Hildebrandt I., Tran C., Halpern B.S., Stout D., Phelps M.E., Weber W.A. (2006). Impact of animal handling on the results of 18f-fdg pet studies in mice. J. Nucl. Med..

[115] Norris D.G. (2003). High field human imaging. J. Magn. Reson. Imag..

[116] Padhani A.R., Liu G., Koh D.M., Chenevert T.L., Thoeny H.C., Takahara T., Dzik-Jurasz A., Ross B.D., Van Cauteren M., Collins D. (2009). Diffusion-weighted magnetic resonance imaging as a cancer biomarker: Consensus and recommendations. Neoplasia.

[117] Cheng H.-L.M., Wallis C., Shou Z., Farhat W.A. (2007). Quantifying angiogenesis in vegf-enhanced tissue-engineered bladder constructs by dynamic contrast-enhanced mri using contrast agents of different molecular weights. J. Magn. Reson. Imag..

[118] Ren J., Huan Y., Wang H., Chang Y.J., Zhao H.T., Ge Y.L., Liu Y., Yang Y. (2008). Dynamic contrast-enhanced mri of benign prostatic hyperplasia and prostatic carcinoma: Correlation with angiogenesis. Clin. Radiol..

[119] Hulka C.A., Edmister W.B., Smith B.L., Tan L., Sgroi D.C., Campbell T., Kopans D.B., Weisskoff R.M. (1997). Dynamic echo-planar imaging of the breast: Experience in diagnosing breast carcinoma and correlation with tumor angiogenesis. Radiology.

[120] Yao W.W., Zhang H., Ding B., Fu T., Jia H., Pang L., Song L., Xu W., Song Q., Chen K. (2011). Rectal cancer: 3 d dynamic contrast-enhanced mri; correlation with microvascular density and clinicopathological features. Radiol. Med..

[121] Haris M., Husain N., Singh A., Awasthi R., Singh Rathore R.K., Husain M., Gupta R.K. (2008). Dynamic contrast-enhanced (dce) derived transfer coefficient (ktrans) is a surrogate marker of matrix metalloproteinase 9 (mmp-9) expression in brain tuberculomas. J. Magn. Reson. Imag..

[122] Orth R.C., Bankson J., Price R., Jackson E.F. (2007). Comparison of single-tracer and dual-tracer pharmacokinetic modeling of dynamic contrast-enhanced mri data using low, medium, and high molecular weight contrast agents. Magn. Reson. Med..

[123] Reitan N.K., Thuen M., Goa P.E., Davies C.D.L. (2010). Characterization of tumor microvascular structure and permeability: Comparison between magnetic resonance imaging and intravital confocal imaging. J. Biomed. Optics.

[124] Mayr N.A., Hawighorst H., Yuh W.T.C., Essig M., Magnotta V.A., Knopp M.V. (1999). Mr microcirculation assessment in cervical cancer: Correlations with histomorphological tumor markers and clinical outcome. J. Magn. Reson. Imag..

[125] Egeland T.A.M., Simonsen T.G., Gaustad J.V., Gulliksrud K., Ellingsen C., Rofstad E.K. (2009). Dynamic contrast-enhanced magnetic resonance imaging of tumors: Preclinical validation of parametric images. Radiat. Res..

[126] Aref M., Chaudhari A.R., Bailey K.L., Aref S., Wiener E.C. (2008). Comparison of tumor histology to dynamic contrast enhanced magnetic resonance imaging-based physiological estimates. Magn. Reson. Imag..

[127] Sinha T.K., Khatib-Shahidi S., Yankeelov T.E., Mapara K., Ehtesham M., Cornett D.S., Dawant B.M., Caprioli R.M., Gore J.C. (2008). Integrating spatially resolved three-dimensional maldi ims with *in vivo* magnetic resonance imaging. Nat. Meth..

[128] Meyer C.R., Moffat B.A., Kuszpit K.K., Bland P.L., McKeever P.E., Johnson T.D., Chenevert T.L., Rehemtulla A., Ross B.D. (2006). A methodology for registration of a histological slide and *in vivo* mri volume based on optimizing mutual information. Mol. Imag..

[129] Zanzonico P.B. (2006). Broad-spectrum multi-modality image registration: From pet, ct, and mri to autoradiography, microscopy, and beyond. Conf. Proc. IEEE Eng. Med. Biol. Soc..

[130] Naish J.H., McGrath D.M., Bains L.J., Passera K., Roberts C., Watson Y., Cheung S., Taylor M.B., Logue J.P., Buckley D.L. (2011). Comparison of dynamic contrast-enhanced mri and dynamic contrast-enhanced ct biomarkers in bladder cancer. Magn. Reson. Med..

[131] Korporaal J.G., van den Berg C.A., van Osch M.J., Groenendaal G., van Vulpen M., van der Heide U.A. (2011). Phase-based arterial input function measurements in the femoral arteries for quantification of dynamic contrast-enhanced (dce) mri and comparison with dce-ct. Magn. Reson. Med..

[132] Kierkels R.G.J., Backes W.H., Janssen M.H.M., Buijsen J., Beets-Tan R.G.H., Lambin P., Lammering G., Oellers M.C., Aerts H.J.W.L. (2010). Comparison between perfusion computed tomography and dynamic contrast-enhanced magnetic resonance imaging in rectal cancer. Int. J. Radiat. Oncol. Biol. Phys..

[133] Ng C.S., Waterton J.C., Kundra V., Brammer D., Ravoori M., Han L., Wei W., Klumpp S., Johnson V.E., Jackson E.F. (2012). Reproducibility and comparison of dce-mri and dce-ct perfusion parameters in a rat tumor model. Tech. Canc. Res. Treat..

[134] Donahue K.M., Weisskoff R.M., Parmelee D.J., Callahan R.J., Wilkinson R.A., Mandeville J.B., Rosen B.R. (1995). Dynamic gd-dtpa enhanced mri measurement of tissue cell volume fraction. Magn. Reson. Med..

[135] Pellerin M., Yankeelov T.E., Lepage M. (2007). Incorporating contrast agent diffusion into the analysis of dce-mri data. Magn. Reson. Med..

[136] Einstein A. (1905). Über die von der molekularkinetischen theorie der wärme geforderte bewegung von in ruhenden flüssigkeiten suspendierten teilchen. Ann. Phys..

[137] Anderson A.W., Xie J., Pizzonia J., Bronen R.A., Spencer D.D., Gore J.C. (2000). Effects of cell volume fraction changes on apparent diffusion in human cells. Magn. Reson. Imag..

[138] Yankeelov T.E., Lepage M., Chakravarthy A., Broome E.E., Niermann K.J., Kelley M.C., Meszoely I., Mayer I.A., Herman C.R., McManus K. (2007). Integration of quantitative dce-mri and adc mapping to monitor treatment response in human breast cancer: Initial results. Magn. Reson. Imag..

[139] Mills S.J., Soh C., Rose C.J., Cheung S., Zhao S., Parker G.J.M., Jackson A. (2010). Candidate biomarkers of extravascular extracellular space: A direct comparison of apparent diffusion coefficient and dynamic contrast-enhanced mr imaging-Derived measurement of the volume of the extravascular extracellular space in glioblastoma multiforme. Am. J. Neuroradiol..

[140] Arlinghaus L.R., Li X., Rahman A.R., Welch E.B., Xu L., Gore J.C., Yankeelov T.E. (2011). On the relationship between the apparent diffusion coefficient and extravascular extracellular volume fraction in human breast cancer. Magn. Reson. Imag..

[141] Dunphy M.P.S., Lewis J.S. (2009). Radiopharmaceuticals in preclinical and clinical development for monitoring of therapy with pet. J. Nucl. Med..

[142] Dearling J., Lewis J., Mullen G., Welch M., Blower P. (2002). Copper bis (thiosemicarbazone) complexes as hypoxia imaging agents: Structure-activity relationships. J. Biol. Inorg. Chem..

[143] Lewis J.S., Welch M.J. (2001). Pet imaging of hypoxia. Q. J. Nucl. Med. Mol. Imag..

[144] Cho H., Ackerstaff E., Carlin S., Lupu M.E., Wang Y., Rizwan A., O’Donoghue J., Ling C.C., Humm J.L., Zanzonico P.B. (2009). Noninvasive multimodality imaging of the tumor microenvironment: Registered dynamic magnetic resonance imaging and positron emission tomography studies of a preclinical tumor model of tumor hypoxia. Neoplasia.

[145] Coleman C.N. (1988). Hypoxia in tumors: A paradigm for the approach to biochemical and physiologic heterogeneity. J. Natl. Canc. Inst..

[146] Jansen J.F., Schoder H., Lee N.Y., Wang Y., Pfister D.G., Fury M.G., Stambuk H.E., Humm J.L., Koutcher J.A., Shukla-Dave A. (2010). Noninvasive assessment of tumor microenvironment using dynamic contrast-enhanced magnetic resonance imaging and 18 f-fluoromisonidazole positron emission tomography imaging in neck nodal metastases. Int. J. Radiat. Oncol. Biol. Phys..

[147] Shaw R.J. (2006). Glucose metabolism and cancer. Curr. Opin. Cell. Biol..

[148] Metz S., Ganter C., Lorenzen S., van Marwick S., Herrmann K., Lordick F., Nekolla S.G., Rummeny E.J., Wester H.J., Brix G. (2010). Phenotyping of tumor biology in patients by multimodality multiparametric imaging: Relationship of microcirculation, αvβ3 expression, and glucose metabolism. J. Nucl. Med..

[149] Partridge S.C., Vanantwerp R.K., Doot R.K., Chai X., Kurland B.F., Eby P.R., Specht J.M., Dunnwald L.K., Schubert E.K., Lehman C.D. (2010). Association between serial dynamic contrast-enhanced mri and dynamic 18 f-fdg pet measures in patients undergoing neoadjuvant chemotherapy for locally advanced breast cancer. J. Mag. Reson. Imag..

[150] Huang J., Chunta J.L., Amin M., Lee D.Y., Grills I.S., Wong C.Y., Yan D., Marples B., Martinez A.A., Wilson G.D. (2012). Detailed characterization of the early response of head-neck cancer xenografts to irradiation using (18) f-fdg-pet imaging. Int. J. Radiat. Oncol. Biol. Phys..

[151] Mani S., Chen Y., Arlinghaus L., Li X., Chakravarthy A., Bhave S., Welch E.B., Levy M., Yankeelov T.E. (2011). Early prediction of the response of breast tumors to neoadjuvant chemotherapy using quantitative mri and machine learning. AMIA Annu. Symp. Proc..

[152] Cheebsumon P., Velasquez L., Hoekstra C., Hayes W., Kloet R., Hoetjes N., Smit E., Hoekstra O., Lammertsma A., Boellaard R. (2011). Measuring response to therapy using fdg pet: Semi-quantitative and full kinetic analysis. Eur. J. Nucl. Med. Mol. Imag..

[153] Braren R., Altomonte J., Settles M., Neff F., Esposito I., Ebert O., Schwaiger M., Rummeny E., Steingoetter A. (2011). Validation of preclinical multiparametric imaging for prediction of necrosis in hepatocellular carcinoma after embolization. J. Hepatol..

[154] Sala E., Kataoka M.Y., Priest A.N., Gill A.B., McLean M.A., Joubert I., Graves M.J., Crawford R.A.F., Jimenez-Linan M., Earl H.M. (2012). Advanced ovarian cancer: Multiparametric mr imaging demonstrates response- and metastasis-specific effects. Radiology.

[155] Brix G., Semmler W., Port R.E., Schad L.R., Layer G., Lorenz W.J. (1991). Pharmacokinetic parameters in cns gd-dtpa enhanced mr imaging. J. Comput. Assist. Tomo..

[156] Larsson H.B.W., Stubgard M., Frederiksen J.L., Jensen M., Henriksen O., Paulson O.B. (1990). Quantitation of blood-brain barrier defect by magnetic resonance imaging and gadolinium-dtpa in patients with multiple sclerosis and brain tumors. Magn. Reson. Med..

[157] Tofts P.S., Kermode A.G. (1991). Measurement of the blood-brain barrier permeability and leakage space using dynamic mr imaging. 1. Fundamental concepts. Magn. Reson. Med..

[158] Donahue M., Blakeley J., Zhou J., Pomper M., Laterra J., van Zijl P. (2008). Evaluation of human brain tumor heterogeneity using mutliple t 1-based mri signal weighting approaches. Magn. Reson. Med..

[159] Jia G., O’Dell C., Heverhagen J., Xang X., Liang J., Jacko R., Sammet S., Pellas T., Cole P., Knopp M.V. (2008). Colorectal liver metastases: Contrast agent diffusion coefficient for quantification of contrast enhancement heterogeneity at mr imaging. Radiology.

[160] Landis C., Li X., Telang F., Coderre J., Micca P., Rooney W., Latour L., Vetek G., Palyka I., Springer C.S. (2000). Determination of the mri contrast agent concentration time course *in vivo* following bolus injection: Effect of equilibrium transcytolemmal water exchange. Magn. Reson. Med..

[161] Yankeelov T.E., Rooney W.D., Huang W., Dyke J.P., Li X., Tudorica A., Lee J.H., Koutcher J.A., Springer C.S. (2005). Evidence for shutter-speed variation in cr bolus-tracking studies of human pathology. NMR Biomed..

[162] Yankeelov T.E., Rooney W.D., Li X., Springer C.S. (2003). Variation of the relaxographic “shutter-speed” for transcytolemmal water exchange affects the cr bolus-tracking curve shape. Magn. Reson. Med..

[163] Li X., Huang W., Yankeelov T.E., Tudorica A., Rooney W.D., Springer C.S. (2005). Shutter-speed analysis of contrast reagent bolus-tracking data: Preliminary observations in benign and malignant breast disease. Magn. Reson. Med..

[164] Zhou R., Pickup S., Yankeelov T.E., Springer C.S., Glickson J.D. (2004). Simultaneous measurement of arterial input function and tumor pharmacokinetics in mice by dynamic contrast enhanced imaging: Effects of transcytolemmal water exchange. Magn. Reson. Med..

[165] Donahue K.M., Weisskoff R.M., Burstein D. (1997). Water diffusion and exchange as they influence contrast enhancement. J. Magn. Reson. Imag..

[166] McConnell H.M. (1958). Reaction rates by nuclear magnetic resonance. J. Chem. Phys..

[167] Woessner D.E. (1961). Nuclear transfer effects in nuclear magnetic resonance pulse experiments. J. Chem. Phys..

[168] Huang W., Tudorica L.A., Li X., Thakur S.B., Chen Y., Morris E.A., Tagge I.J., Korenblit M.E., Rooney W.D., Koutcher J.A. (2011). Discrimination of benign and malignant breast lesions by using shutter-speed dynamic contrast-enhanced mr imaging. Radiology.

